# Adolescent ICT use profiles and creative thinking: evidence from 42 countries in PISA 2022

**DOI:** 10.3389/fpsyg.2026.1950010

**Published:** 2026-10-29

**Authors:** Can Meşe, Muhammet Serdar Başçıl

**Affiliations:** 1 Department of Digital Game Design, Faculty of Communication, Kahramanmaraş Istiklal University, Kahramanmaraş, Türkiye; 2 Department of Electrical and Electronics Engineering, Faculty of Technology, Selçuk Universitesi, Konya, Türkiye

**Keywords:** creative thinking, ICT use, latent profile analysis, PISA 2022, random forest

## Abstract

Research on information and communication technology (ICT) use and educational outcomes has reported positive, negative, and null associations, and little is known about creative thinking, which Programme for International Student Assessment (PISA) assessed for the first time in 2022. This study examined how profiles of ICT use relate to creative thinking and whether profile membership predicts creative thinking beyond the dimensions of use from which the profiles are derived. Using data from a large international sample of PISA 2022 students, latent profile analysis of the six Organisation for Economic Co-operation and Development (OECD) dimensions of ICT use identified four profiles that differed mainly in the overall level of use. Creative thinking was compared across profiles using all plausible values, replicate weights and a bias-adjusted three-step approach, and random forest models compared the predictive value of the profiles and the dimensions. Students with below-average use scored highest, followed by those with above-average use, whereas students with very low and very high use scored lowest. The low scores at the two ends had different correlates: adjustment for socioeconomic status and gender substantially reduced the gap for very low use but left the gap for very high use almost unchanged. In the random forest models, the six dimensions predicted creative thinking better than profile membership did. Partial dependence plots showed higher predicted scores with more school activities outside the classroom and lower predicted scores with high weekday leisure use and more support or feedback via ICT. The findings suggest that the association between ICT use and creative thinking is not linear, and that profiles based on the level of use are useful for describing groups of students but carry less information than the dimensions of use.

## 1 Introduction

Information and Communication Technologies (ICT) have recently become a part of daily life for adolescents in most countries, changing the ways young people learn, socialize, and spend their spare time. Policy frameworks such as the Organisation for Economic Co-operation and Development (OECD) Learning Compass 2030 regard digital skills as a core element of education (OECD, 2019), and the European Commission's DigCompEdu framework has outlined the digital competences for educators (Redecker, 2017). Moreover, many national curricula have added new digital competences to compulsory schooling (Uzunboylu et al., 2017). Because most 15-year-olds, the target population of the Programme for International Student Assessment (PISA), now have access to digital devices, research increasingly emphasizes students' patterns of technology use and their implications for educational outcomes (Hristov et al., 2025; Kim and Liou, 2026).

Existing research on ICT and education has produced mixed findings, suggesting that more ICT use does not necessarily lead to better educational outcomes. Cross-national analyses of PISA data have reported both positive and negative associations between computer use and achievement in mathematics, reading, and science (Biagi and Loi, 2013; Hu et al., 2018; Petko et al., 2017; Spiezia, 2010; Zhang and Liu, 2016), and a meta-analysis of 48 studies found a positive overall correlation between digital device use and adolescents' academic performance, but educational use was positively related to performance, whereas use for entertainment, socializing and gaming, and longer use in general, were negatively related (Wang et al., 2024). Part of the inconsistency may stem from how ICT use has been operationalized in these studies. Most analyses treat technology use as a single continuum from low to high frequency, without separating the purposes and settings of use (Kim and Liou, 2026). Under this approach, a student who uses ICT mainly for schoolwork and one who spends the same amount of time playing video games end up in the same high-use category. Yet the amount and the purpose of use need not relate to outcomes in the same way. In Dutch PISA data, for example, students with moderate ICT use at school and for schoolwork had the highest reading scores, whereas leisure use was negatively related to reading (Gubbels et al., 2020). A single frequency measure, or a model that assumes a linear relationship, can hide such patterns. Moreover, most of this research has focused on achievement in mathematics, reading, and science, and much less is known about creative thinking, the capacity to generate, evaluate, and improve ideas (OECD, 2024b), and whether it relates to ICT use in the same way. The PISA 2022 cycle, which included an assessment of creative thinking given to over 140,000 students in more than 60 countries (Barbot and Kaufman, 2025; OECD, 2024b), provides an opportunity to investigate the relationship between ICT use and creative thinking at a large scale.

The aim of the current study is to examine how patterns of adolescent ICT use relate to creative thinking in PISA 2022. To address this aim, the study combines person-centered profiling, survey-weighted inference, and machine learning. Using the PISA 2022 international dataset, it identifies latent profiles of ICT use across the six ICT use dimensions of the PISA 2022 ICT framework, which cover school-related use in and outside the classroom and leisure use on weekdays and weekends (OECD, 2023a). It then compares creative thinking across these profiles, within countries and after adjustment for socioeconomic status and gender, and uses random forest models to examine whether profile membership adds predictive information beyond the six dimensions from which it was derived, students' background, and ICT availability. Because the number and shape of the profiles were not known in advance, the analyses are exploratory, and no directional hypotheses were specified. The following section provides a detailed explanation of the theoretical and empirical rationale for this design. The study seeks to answer the following research questions:

1. **RQ1:** What latent ICT use profiles can be identified across the six ICT use dimensions of PISA 2022?
2. **RQ2:** How prevalent are these profiles, and how do they differ in background and ICT availability?
3. **RQ3:** How does creative thinking differ across the profiles, within countries and after adjustment for socioeconomic status (ESCS) and gender?
4. **RQ4:** How important is the ICT use profile for predicting creative thinking compared with ICT use dimensions, background, and ICT availability?

## 2 Theoretical framework and literature review

### 2.1 Creative thinking and ICT use in PISA 2022

PISA 2022 introduced a large-scale assessment of creative thinking, the largest study of adolescent creativity conducted to date, covering over 140,000 students in more than 60 countries (Barbot and Kaufman, 2025). The OECD framework defines creative thinking as the competence to engage productively in generating, evaluating, and improving ideas that can result in original and effective solutions, advances in knowledge, or impactful expressions of imagination (OECD, 2024b). Students completed open-ended cognitive tasks across four content domains: written expression, in which students produced short texts such as captions, story ideas, or dialogues; visual expression, in which they created or modified visual designs; scientific problem-solving, in which they proposed hypotheses or new or improved methods for solving problems; and social problem-solving, in which they proposed solutions to problems affecting groups of people or society at large (Barbot and Kaufman, 2025; OECD, 2024b). Responses were scored using rubrics that assessed originality and appropriateness rather than a single correct answer. A rotated-booklet design meant that only a subset of sampled students took the creative thinking module, which has implications for sample composition and analytical strategy (Rutkowski and Rutkowski, 2025; Selva and Heine, 2026).

Early methodological work on the PISA 2022 creative thinking data has raised both opportunities and cautions. Barbot and Kaufman (2025) noted that the aggregated scoring approach and the complex sampling design present methodological challenges, and that careless secondary analyses risk producing fragmented or inconsistent results. At the measurement level, Kraus et al. (2026) applied item-level machine learning to examine construct validity and found that the three assessed creative thinking processes (generating creative ideas, generating diverse ideas, and evaluating and improving ideas) behaved more similarly than the theoretical framework would predict. Academic competences in mathematics, reading and science were among the strongest predictors of item scores, whereas self-reported creativity and creative activities were of limited relevance. These findings are relevant to the present study because they suggest that creative thinking scores also reflect general academic competence, which may itself be related to how students use digital technology.

Several empirical studies have examined creative thinking using the PISA 2022 data. Across countries, creative thinking performance varies substantially, with ecological and systemic factors (educational policy orientation, cultural emphasis on divergent thinking) helping to explain why some high-performing countries in traditional domains rank differently on creative thinking (Burakgazi and Reiss, 2025). At the student level, growth mindset, ICT self-efficacy, and environmental support have been identified as predictors (Zeng, 2025), and machine learning analyses in East Asian subsamples have placed socio-ecological and motivational factors among the strongest determinants (Chen et al., 2026; Wu et al., 2026). Goecke et al. (2026), analyzing data from 62 countries, found that female students consistently outperformed males and that socio-economic status predicted creative thinking at a magnitude comparable to its effects on mathematics and reading. Tian X. et al. (2026) used Shapley value decomposition with PISA 2022 Asia-Pacific data and found that ICT self-efficacy substantially weakened the negative association between disadvantaged family background and creative thinking, pointing to how students use technology, not merely whether they have access, as an equity-relevant factor. Fu et al. (2026), working from ecological systems theory with the same dataset, showed that creative personality positively predicted creative thinking, but that creative peer and family environments negatively moderated this relationship.

The PISA 2022 ICT questionnaire describes students' use of ICT by its purpose and setting (OECD, 2023a). Six indices capture how often students use ICT in subject lessons (ICTSUBJ), in enquiry-based learning activities (ICTENQ), to receive support or feedback (ICTFEED), and for school activities outside the classroom (ICTOUT), and how much time they spend on ICT for leisure on a typical weekday (ICTWKDY) and weekend day (ICTWKEND). These dimensions are not interchangeable. School-related and leisure use involve different activities, and in PISA data they have been related to achievement in different directions (Gubbels et al., 2020; Melnikova et al., 2026). Research on social media makes a similar distinction between passive browsing and active use (Dienlin and Johannes, 2020; Verduyn et al., 2022), although active use in that literature refers to interacting with others rather than to creating content. Moreover, Chu (2025) showed that ICT conditions relate to creative thinking through several configurations at the country level rather than through a single linear effect. Whether students themselves combine different kinds of ICT use, and how such combinations relate to creative thinking, has not been examined at the student level.

Some of the emerging findings are counterintuitive. Kagan et al. (2026) reported a negative correlation between students' engagement in creative extracurricular activities and their creative thinking scores across different countries. In the ICT domain, Cai et al. (2026) used SHapley Additive exPlanations (SHAP)-based interpretable models with data from five East Asian education systems and found that ICT behaviors, including digital content creation, information retrieval, and collaborative communication, predicted creative thinking in largely nonlinear and level-dependent ways. Kaplan et al. (2025) applied propensity score matching across two contrasting educational systems and found a positive association between teachers' integration of digital technology and students' creative thinking. Taken together, these results suggest that the relationship between ICT use and creative thinking is nonlinear and depends on how students use ICT, not only on how much. A single average slope across all students is likely to miss such patterns.

### 2.2 Amount of use and digital inequality

The amount of ICT use is a second reason why a single linear slope may be misleading. According to the displacement hypothesis, time spent with digital media replaces activities that would otherwise support development, so more use should go with poorer outcomes (see Przybylski and Weinstein, 2017). The digital Goldilocks hypothesis instead holds that moderate use is not harmful and may even be beneficial, and that only use beyond a certain point is associated with poorer outcomes. In a preregistered study of 120,115 English adolescents, Przybylski and Weinstein (2017) found such curvilinear links between screen time and mental wellbeing, with lower turning points on weekdays than at weekends. Evidence from PISA points in the same direction: students with moderate ICT use at school and for schoolwork had the highest reading scores, whereas leisure use was negatively associated with reading (Gubbels et al., 2020).

Differences in ICT use, however, are not merely matters of personal preference. As access to digital technology has spread, inequalities have increasingly shifted from access to differences in how the internet is used (van Deursen and van Dijk, 2014). Among students, cultural capital has been linked to educational ICT use but not to leisure use (Ren et al., 2022). Students who rarely use ICT for school purposes may therefore come from less advantaged homes, and a lower creative thinking score at the low end of use may partly reflect socioeconomic background. At the high end, displacement or other characteristics of intensive users may matter more. For this reason, the present study compares creative thinking across profiles before and after adjustment for socioeconomic status and gender, and within socioeconomic quartiles.

### 2.3 Person-centered and predictive approaches

Most research on ICT and education relies on variable-centered designs that estimate the average association of ICT frequency across the student population (Fernández-Gutiérrez et al., 2020; Kim and Liou, 2026). These designs capture general trends, but they assume a uniform relationship for all students, and that assumption becomes questionable when students combine kinds of ICT use in different ways. Person-centered methods identify subgroups of individuals who share similar patterns and test whether subgroup membership is related to different outcomes (Spurk et al., 2020). Latent Profile Analysis (LPA), for instance, has been applied to parental ICT proficiency and adolescents' information literacy (Wu et al., 2020), to screen time trajectories and academic performance (Kaye and Symonds, 2025), and to technology use profiles before and during the COVID-19 pandemic (Kastorff et al., 2023). Hermansen (2023) used latent class analysis in higher education and identified four ICT use-and-production profiles among Danish university college students, showing that different kinds of use can be empirically separated. In PISA data, profiles of students' ICT use have often differed mainly in the overall level of use, and the group with the most intensive use has tended to be small and to score lower in the achievement domains examined (Karakoyun and Başaran, 2022; Kim and Kim, 2023; Xiao and Sun, 2022). Among existing work, Tan et al. (2026) are most relevant to the present study, having used multilevel LPA on PISA 2022 data to characterize ICT use profiles and their associations with academic and social-emotional outcomes. Their study did not examine creative thinking, nor did it test whether profile membership carries information beyond the ICT use dimensions from which the profiles were derived.

Random forest and related machine learning methods have gained traction in PISA research as tools for identifying predictors of mathematics, reading, and science performance (Lezhnina and Kismihók, 2022; Öz et al., 2025; Zheng et al., 2025; Zhu et al., 2025). These methods are useful because they accommodate large numbers of correlated predictors and nonlinear associations without requiring their form to be specified in advance (Breiman, 2001), which makes them well suited for large-scale survey data. They also allow different sets of predictors to be compared within the same framework.

Taken together, this body of work suggests that the relationship between ICT use and educational outcomes depends on both the amount and the purpose of use, even though most large-scale analyses still treat technology use as a single frequency measure. Person-centered methods can describe how students combine different kinds of use, but a profile is a summary of the dimensions it is built from. If these dimensions relate to an outcome in different directions, a profile defined mainly by the level of use may carry less information than the dimensions themselves. The present study therefore uses LPA to identify ICT use profiles, compares creative thinking across them, and uses random forest models to examine whether profile membership predicts creative thinking beyond the six ICT use dimensions, students' background, and ICT availability.

## 3 Methods

### 3.1 Research design

This study employed a correlational research design within the quantitative research tradition to examine how students' ICT use profiles relate to their creative thinking performance in PISA 2022. Correlational research is used to examine the degree and direction of the relationship between two or more variables without manipulating them (Fraenkel et al., 2011). This design was appropriate for the present study because the aim was to identify how the patterns of ICT use relate to creative thinking outcomes in a large-scale secondary dataset. The analysis proceeded in four stages: confirmatory factor analysis of the ICT use items, LPA to identify ICT use profiles, survey-weighted group comparisons to test creative thinking differences across profiles, and a random forest model to assess the relative predictive contribution of profile membership compared with the ICT use factors it was derived from, students' background, and ICT availability.

### 3.2 Data and sample

The PISA 2022 evaluation of 15-year-olds enrolled in schools across 81 countries and economies involved approximately 690,000 students (OECD, 2024b). PISA 2022 included creative thinking as a new assessment domain. Due to the rotated booklet design, in which only about 29% of students received a creative thinking test form, not every student received every assessment module (Selva and Heine, 2026; Zieger et al., 2022).

The data were obtained from the PISA 2022 dataset administered by OECD. We merged two public-use files at the student level: the student questionnaire file (613,744 students, 80 countries and economies) and the creative thinking cognitive file (499,843 students, 63 countries and economies). The analytical pipeline proceeded through six sequential filtering stages (Figure 1) of data from PISA 2022 database (OECD, 2024b). The questionnaire file provides the ICT familiarity items, background variables, the final student weight and the 80 replicate weights; the cognitive file provides the 10 plausible values of creative thinking and the scored task responses. Records were matched on country, school and student identifiers. Each record in the cognitive file matched exactly one questionnaire record, and no duplicate identifiers were found.

**Figure 1:**
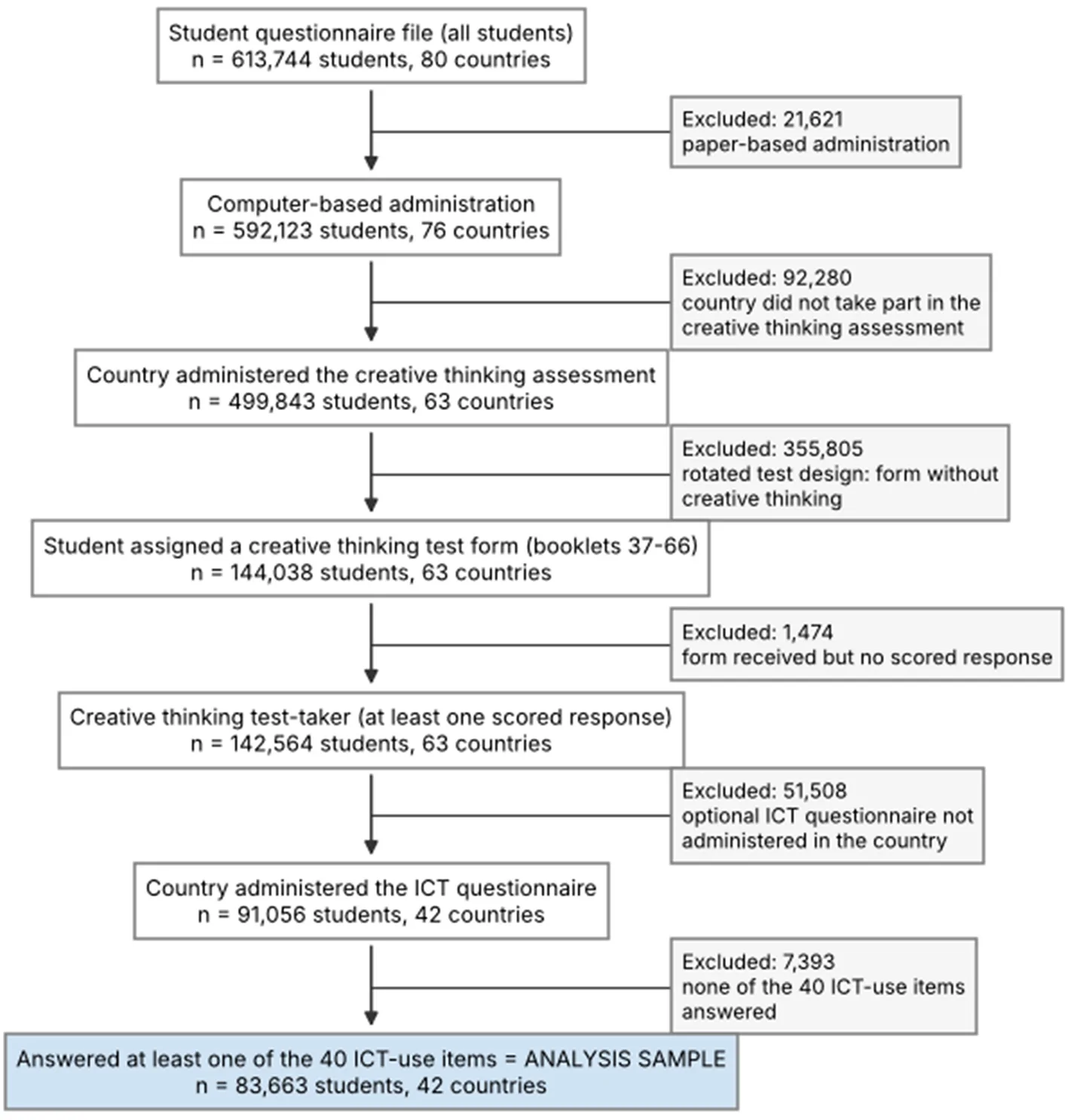
Flow diagram of sample selection.

The merged data, firstly restricted to the data to computer-based administration, excluded 21,621 students in four countries (*n* = 592,123), and to the 63 countries that administered the creative thinking assessment, excluded a further 92,280 (*n* = 499,843). Under the rotated design, 144,038 of these students from 63 countries were assigned a creative thinking test form (booklets 37–66). In the next step, we kept those with at least one scored response (*n* = 142,564). The 1,474 students who received such a form but gave no scorable response were excluded, because their plausible values would rest entirely on the background model. The ICT familiarity questionnaire is an optional PISA instrument and was administered in 42 of the 63 countries, so the 51,508 test-takers in the remaining 21 countries could not be included (*n* = 91,056). Finally, 7,393 students (8.1%) who answered none of the 40 ICT-use items were excluded; all others were kept regardless of how many items they answered.

According to Table 1, the analysis sample comprised 83,663 students in 42 countries and economies, that is, 58.7% of all creative thinking test-takers and 91.9% of the test-takers in countries that administered the ICT questionnaire. Country samples ranged from 785 to 8,256 students, and within countries the sample covered 76.5% to 100% of test-takers (median 93.8%; Supplementary Table S1).

**Table 1:** Sample flow from the PISA 2022 file to the analysis sample.

Step	Criterion	Students	Countries	Excluded	Reason for exclusion
0	PISA 2022 student questionnaire file	613,744	80	—	—
1	Computer-based administration	592,123	76	21,621	Paper-based administration (GTM, KHM, PRY, VNM)
2	Country administered the creative thinking assessment	499,843	63	92,280	13 countries did not administer the assessment
3	Assigned a creative thinking test form (booklets 37–66)	144,038	63	355,805	Rotated test design (form without creative thinking)
4	At least one scored creative thinking response	142,564	63	1,474	Form received, no scorable response
5	Country administered the ICT questionnaire	91,056	42	51,508	Optional questionnaire not administered in 21 countries
6	At least one of the 40 ICT-use items answered (analysis sample)	83,663	42	7,393	No ICT-use item answered

The countries excluded at Step 2 were ARG, AUT, CHE, GBR, GEO, IRL, JPN, KSV, MNE, NOR, SWE, TUR, and USA. At Step 5 they were ARE, CAN, COL, FRA, IDN, JAM, MDA, MEX, MKD, MNG, NLD, NZL, PER, PHL, PRT, PSE, QAT, QAZ, SLV, SRB, and UZB.

### 3.3 Measures and variables

#### 3.3.1 Creative thinking items

The dependent variable consisted of scores derived from the PISA 2022 Creative Thinking cognitive assessment, the first large-scale international measure of adolescent creative thinking, covering over 140,000 students in more than 60 countries (Barbot and Kaufman, 2025; OECD, 2024b). The PISA 2022 framework defines creative thinking as the competence to engage productively in generating, evaluating, and improving ideas. The assessment measures three cognitive processes: generating diverse ideas, generating creative ideas, and evaluating and improving ideas. Students completed open-ended tasks across four content domains: written expression, visual expression, scientific problem-solving, and social problem-solving. Responses were scored using rubric-based criteria that evaluated the originality and appropriateness of the ideas produced. Rather than reporting a single raw score, PISA uses Item Response Theory (IRT)-based plausible values: 10 plausible values (PV1CRTH_NC through PV10CRTH_NC) per student (Rutkowski and Rutkowski, 2025). Scores are reported on a 0–60 scale; following PISA guidelines, the 10 plausible values were analyzed separately and the results combined (see Section 3.5).

#### 3.3.2 ICT use

To measure students' frequency of ICT use, we selected 40 items covering the six dimensions of ICT use in the PISA 2022 ICT framework (OECD, 2023a): subject-related use during lessons (ICTSUBJ; IC173, 4 items), use in enquiry-based learning activities (ICTENQ; IC174, 10 items), support or feedback via ICT (ICTFEED; IC175, 4 items), use for school activities outside the classroom (ICTOUT; IC176, 8 items), and leisure use on a typical weekday (ICTWKDY; IC177, 7 items) and weekend day (ICTWKEND; IC178, 7 items). IC173–IC176 were rated on five-point frequency scales, and IC177 and IC178 on a six-point time scale (1 = no time at all, 6 = more than 7 h a day). In IC173, the option “I do not have this subject” (code 6) does not express a frequency and was treated as missing (OECD, 2024a). In IC177 and IC178, code 6 is the highest valid category and was retained. PISA missing-data codes were set to missing.

The six-factor structure was tested by confirmatory factor analysis in *Mplus* via the *MplusAutomation* R package with robust maximum likelihood (MLR), treating items as continuous, with missing responses handled by full information maximum likelihood (FIML) (Section 3.4). The model was estimated without weights, so that the pooled model was not dominated by the most populous countries. Because the weekday and weekend items describe the same seven activities, the residuals of each weekday–weekend item pair were allowed to covary. The parameters weren't added on the basis of modification indices. The model fitted acceptably [χ^2^ = 136,283, *df* = 718; CFI = 0.905; TLI = 0.897; RMSEA = 0.048, 90% CI (0.047, 0.048); SRMR = 0.050].

Standardized loadings ranged from 0.46 to 0.85, except for use in computer science lessons (IC173Q04; 0.40), which 17% of students reported not having; the item was retained to keep the OECD structure. Composite reliability ranged from 0.80 to 0.93 and average variance extracted from 0.496 to 0.631. Weekday and weekend leisure use were highly correlated (*r* = 0.85; HTMT = 0.93), so their discriminant validity was limited; the other factor correlations ranged from 0.22 to 0.61. Treating items as ordinal (WLSMV) gave similar fit (CFI = 0.929; robust CFI = 0.894; robust RMSEA = 0.069). Weighting by senate weights with stratum and school clustering left fit and loadings virtually unchanged (CFI = 0.907; maximum loading difference = 0.018).

Factor scores from this model were used as indicators in LPA. When a student had answered none of the items of a factor (3.6%−9.0% of students per factor), that score was set to missing rather than predicted from the other factors, because such a score would carry no information about that dimension itself. These missing indicator values were handled by FIML in the LPA.

#### 3.3.3 Background and ICT availability

Gender, socioeconomic status (ESCS; a composite of parental education, parental occupation, and home possessions; OECD, 2023b) and immigrant background were taken from the student questionnaire. ICT availability was measured by two OECD indices, availability outside school (ICTHOME) and at school (ICTSCH) (OECD, 2024a). These variables were used to describe the profiles, in the adjusted and subgroup comparisons, and as predictors in the random forest models. All variables are listed in Supplementary Table S2.

### 3.4 Missing data

In the analysis sample, the proportion of missing responses per item ranged from 5.5% to 21.7% for IC173 and from 6.4% to 11.3% for the other blocks (Supplementary Table S3). The higher rate for IC173 came mainly from the option “I do not have this subject,” most often chosen for computer science lessons (IC173Q04). In most blocks, students skipped some items more often than the whole block (e.g., IC174: 10.4% vs. 4.1%); only in IC175 and IC178 was skipping the whole block more common (5.5% and 9.0%). In total, 48,031 students (57.4%) answered all 40 items.

Rather than deleting students with incomplete responses, we retained all students who answered at least one ICT-use item. Missing item responses in the measurement model, and missing factor scores in the latent profile models, were handled by full information maximum likelihood (FIML), which uses all available responses and gives unbiased estimates when data are missing at random (MAR). FIML is recommended for item- and factor-level analyses of the PISA 2022 creative thinking data, whereas plausible values are intended for population-level comparisons (Selva and Heine, 2026). Creative thinking was therefore not used as a profile indicator and entered only the between-profile comparisons.

Because missing responses may be related to the outcome, we compared the 83,663 included students with the 7,393 excluded students from the same 42 countries (Table 2). Differences in gender and immigrant background were negligible (SMD ≤ 0.06), but excluded students had lower ESCS (SMD = 0.20) and lower creative thinking scores (SMD = 0.34; median within-country SMD = 0.31). The excluded group is small, but it is not a random subset, so the findings should be generalized with caution to students who did not respond to the ICT questionnaire. Test-takers in the 21 countries without the ICT questionnaire also scored lower (weighted means 21.86 vs. 27.10), so the findings refer only to the 42 countries and economies that administered it.

**Table 2:** Students included in and excluded from the analysis sample.

Variable	Included (*n* = 83,663)	Excluded (*n* = 7,393)	SMD
Creative thinking (mean of 10 plausible values)	27.56	23.47	0.34
Female (proportion)	0.51	0.50	0.02
ESCS	−0.50	−0.75	0.20
Immigrant background (proportion)	0.07	0.06	0.06

Students in the 42 countries that administered the ICT questionnaire. Estimates weighted by the final student weight (W_FSTUWT). SMD = difference in weighted means divided by the pooled weighted standard deviation; for creative thinking, SMDs were computed for each plausible value and averaged (range 0.32–0.36; median within-country SMD = 0.31, 41 countries). Missing covariate values among included/excluded students: ESCS 2,234/1,751; gender 3/0; immigrant background 2,739/1,959. ESCS was missing for 23.7% of the excluded students.

### 3.5 Data analysis

Analyses were run in R 4.5.1 and Mplus 8.7 via MplusAutomation v1.3 (Hallquist and Wiley, 2018). The measurement and profile models were estimated without weights. For all comparisons of creative thinking and background across profiles, each plausible value was analyzed separately with the final student weight, standard errors were estimated with Fay's balanced repeated replication (80 replicate weights, *k* = 0.5), and results were combined with Rubin's rules (OECD, 2024a; Rubin, 1987).

In the second stage, LPA (Spurk et al., 2020) was conducted in *Mplus* via the *MplusAutomation* R package (Hallquist and Wiley, 2018; Muthén and Muthén, 2017), using the six factor scores (Section 3.3.2) as indicators, with means free and variances equal across profiles. Because weekday and weekend leisure use describe the same activities (*r* = 0.85), their covariance was estimated and held equal across profiles; a local independence model served as comparison. Models with one to eight profiles were estimated with 400 random starts and 100 final-stage optimizations (50 initial iterations), and the best loglikelihood was replicated in each. The number of profiles was chosen from the elbow in Bayesian information criterion (BIC), consistent Akaike information criterion (CAIC), approximate weight of evidence (AWE), and integrated completed likelihood BIC (ICL-BIC), average posterior probabilities above 0.70 and odds of correct classification above 5 for every profile (Masyn, 2013; Nagin, 2005; Nylund-Gibson and Choi, 2018), profile size, and whether profiles persisted when one was added. Likelihood ratio tests (VLMR, LMR, and BLRT; McLachlan and Peel, 2000) are reported but were not decisive, as with this sample size they keep favoring more profiles (Nylund et al., 2007). The three-profile solution is reported as a sensitivity analysis (Supplementary Table S6).

In the third stage, students were assigned to their most likely profile, and classification error was corrected with the BCH method (Bakk and Vermunt, 2016; Bolck et al., 2004; Vermunt, 2010), which weights each student by the inverse of the classification error probabilities so that profile means are not biased toward each other; the weights were recomputed for every replicate weight. Creative thinking was compared across profiles with an overall Wald test and Bonferroni-corrected pairwise contrasts (d = mean difference divided by the pooled within-profile SD). The comparison was repeated with country fixed effects, with adjustment for ESCS and gender, within ESCS quartiles and gender groups, and with senate weights rescaled to 5,000 students per country.

Finally, random forests (Breiman, 2001), which allow for nonlinear associations and correlated predictors, were used to compare the predictive value of the profile with that of the six factor scores, background (gender, ESCS, immigrant background) and ICT availability (ICTHOME, ICTSCH). Models were fitted with *ranger* 0.18.0 (Wright and Ziegler, 2017) using 500 trees, mtry = √p rounded down, a minimum node size of 5, and student weights as case weights. Costa Rica, which lacks ESCS, was excluded (*n* = 82,016, 41 countries). Because FIML is not available for random forests, missing predictors were imputed by predictive mean matching (mice 3.19.0; van Buuren and Groothuis-Oudshoorn, 2011), once per plausible value, without the outcome in the imputation model. Accuracy was estimated by five-fold cross-validation with schools assigned to folds within countries, and predictor sets were compared by the change in weighted R^2^. Permutation importance was computed in the held-out folds, and partial dependence plots show the shape of associations (Friedman, 2001). To account for classification uncertainty, the modal profile was also replaced by posterior probabilities and by a profile drawn from each student's posterior. Complete-case results are reported with the random forest results (Section 4.4), and item-level analyses in Supplementary Table S9.

## 4 Findings

### 4.1 ICT use profiles (RQ1)

To answer RQ1, latent profile models with one to eight profiles were estimated from the six ICT use factor scores (ICTSUBJ, ICTENQ, ICTFEED, ICTOUT, ICTWKDY, and ICTWKEND) of the 83,663 students in the analysis sample. Fit statistics for all solutions are reported in Table 3.

**Table 3:** Fit of latent profile models with one to eight profiles.

K	LL	**Par**.	**BIC**	CAIC	AWE	ICL-BIC	Entropy	Smallest (%)	Min. AvePP	Min. OCC	VLMR p	LMR p
1	−504,634.1	13	1,009,416	1,009,429	1,009,602	1,009,416	—	100.0	—	—	—	—
2	−460,360.2	20	920,947	920,967	921,234	951,028	0.740	47.3	0.922	10.8	0.333	0.333
3	−443,877.2	27	888,060	888,087	888,448	930,223	0.770	19.9	0.884	7.1	< 0.001	< 0.001
**4**	**−437,563.6**	**34**	**875,513**	**875,547**	**876,000**	**933,569**	**0.749**	**11.0**	**0.830**	**8.1**	**< 0.001**	**< 0.001**
5	−434,629.6	41	869,724	869,765	870,312	929,645	0.777	1.3	0.827	8.0	< 0.001	< 0.001
6	−431,030.7	48	862,605	862,653	863,294	924,952	0.792	1.5	0.825	7.9	< 0.001	< 0.001
7	−428,182.7	55	856,989	857,044	857,777	928,605	0.780	1.6	0.777	7.1	< 0.001	< 0.001
8	−425,321.6	62	851,346	851,408	852,235	937,171	0.753	1.5	0.743	7.0	< 0.001	< 0.001

The selected solution is shown in bold. LL, loglikelihood; Par, number of free parameters; BIC, Bayesian information criterion; CAIC, consistent Akaike information criterion; AWE, approximate weight of evidence; ICL-BIC, integrated completed likelihood BIC; Smallest, smallest profile (% of the sample, model-estimated); AvePP, average posterior probability of the assigned profile; OCC, odds of correct classification; VLMR and LMR, Vuong–Lo–Mendell–Rubin and Lo–Mendell–Rubin likelihood ratio tests (K vs. K – 1). Bootstrapped likelihood ratio test (BLRT) for four vs. three profiles: *p* < 0.05 (none of 20 bootstrap draws exceeded the observed value). Fit of the local independence model: Supplementary Table S4.

For every number of profiles, the model with a weekday–weekend covariance fitted considerably better than the local independence model (e.g., ΔBIC = −64,616 for four profiles), so it was used throughout. BIC, CAIC, and AWE fell steeply up to four profiles (BIC decreases of 88,469, 32,887, and 12,547 for two, three, and four profiles) and much less thereafter (5,616 to 7,119 per additional profile), which places the elbow at four profiles (see Figure 2). ICL-BIC, which additionally penalizes classification uncertainty, was slightly lower for three than for four profiles (930,223 vs. 933,569) and reached its minimum at six profiles. The likelihood ratio tests favored each larger model from three profiles onward (VLMR and LMR *p* < 0.001; BLRT *p* < 0.05 for four vs. three profiles), as expected with more than 80,000 students.

**Figure 2:**
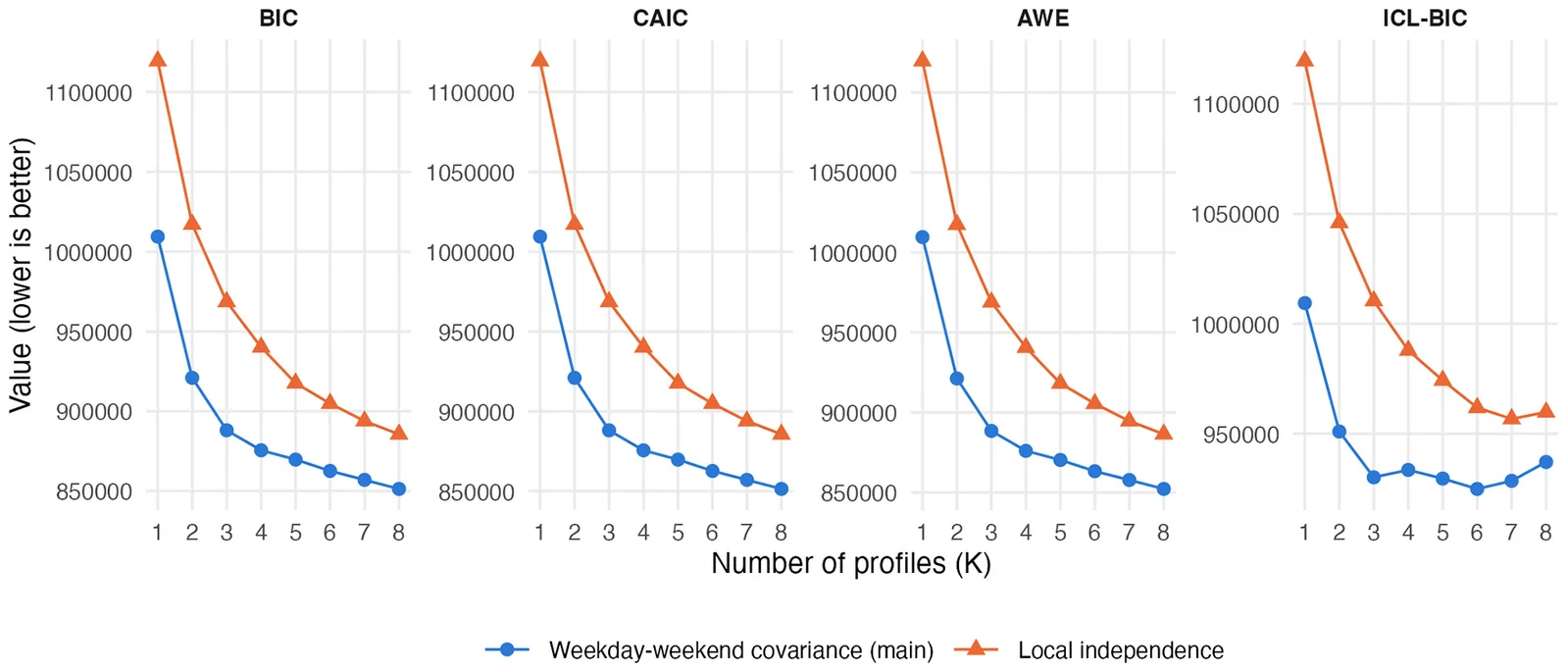
Information criteria for latent profile models with one to eight profiles.

In the four-profile solution, average posterior probabilities ranged from 0.830 to 0.881 and odds of correct classification from 8.1 to 59.5, well above the thresholds of 0.70 and 5. Entropy (0.749) was below the often-cited value of 0.80, and 19.1% of students had no posterior probability above 0.70. The smallest profile comprised 11.0% of the sample.

The four profiles were stable when further profiles were added. In the five-profile solution, 98.0% to 99.1% of the students in each profile were assigned to the corresponding profile, and the additional profile (1.1%) consisted of students with high weekend but average weekday leisure use. The six-profile solution added a similar profile with high weekday use only (1.3%), while the four main profiles kept virtually the same means. Profiles that differed in shape, with high school-related but average leisure use, appeared only with seven or eight profiles, solutions with lower classification accuracy (smallest average posterior probability 0.777 and 0.743; Supplementary Table S5).

Moving from three to four profiles changed all three profiles. The lowest profile split into Profile 1 (60.9% of its students) and Profile 2 (39.1%), the highest split into Profile 4 (65.9%) and Profile 3 (34.1%), and about one third of the middle profile (32.3%) moved to Profile 1. Profiles 1 and 2 differed clearly in socioeconomic status (mean ESCS −0.44 vs. −1.03; SMD = 0.50) and in the share of girls (55.9% vs. 42.4%). Together with the elbow in the information criteria and adequate classification for every profile, this supported retaining four profiles. With three profiles, the association with creative thinking showed the same pattern (Supplementary Table S6).

### 4.2 ICT use profiles and their composition (RQ2)

The four latent profiles are summarized in Table 4 and depicted in Figure 3. Percentages are weighted estimates of profile size, and *n* is the number of students assigned to each profile.

**Table 4:** Characteristics of the four ICT use profiles (*N* = 83,663).

**Characteristic**	Profile 1	Profile 2	Profile 3	Profile 4
	Below-average use	Very low use	Above-average use	Very high use
Weighted share, % (SE)	33.2 (0.3)	15.0 (0.2)	39.0 (0.3)	12.8 (0.2)
Students assigned (*n*)	28,056	8,463	36,571	10,573
Average posterior probability	0.830	0.881	0.857	0.868
***ICT use (profile mean, z)***				
Subject-related use in lessons (ICTSUBJ)	−0.38	−0.81	0.25	0.94
Enquiry-based learning (ICTENQ)	−0.57	−1.39	0.37	1.45
Support or feedback via ICT (ICTFEED)	−0.61	−1.30	0.39	1.51
School activities outside the classroom (ICTOUT)	−0.31	−1.71	0.34	1.21
Leisure use, weekday (ICTWKDY)	−0.33	−0.68	0.18	0.89
Leisure use, weekend (ICTWKEND)	−0.29	−0.66	0.19	0.85
***Background and ICT availability (weighted; SE)***				
Girls, %	55.9 (0.8)	42.4 (1.1)	50.3 (0.7)	48.3 (1.2)
Immigrant background, %	7.4 (0.4)	4.9 (0.4)	7.7 (0.3)	6.4 (0.5)
ESCS, mean	−0.44 (0.02)	−1.03 (0.03)	−0.37 (0.02)	−0.44 (0.03)
ICT availability outside school (ICTHOME), mean	−0.06 (0.02)	−0.11 (0.03)	−0.03 (0.01)	−0.46 (0.04)
ICT availability at school (ICTSCH), mean	−0.10 (0.02)	−0.09 (0.03)	−0.03 (0.02)	−0.37 (0.04)

Profiles are numbered as estimated in Mplus. Weighted shares and background and availability means are BCH-corrected estimates weighted by the final student weight; standard errors (SE) are based on the 80 replicate weights. ICT use means are model-estimated profile means of the factor scores, expressed as z = (profile mean – sample mean) / sample SD (unweighted). Students assigned = number of students whose most likely profile is the given profile. ESCS, index of economic, social and cultural status. ICT availability indices as provided by the OECD; higher values indicate more available resources.

**Figure 3:**
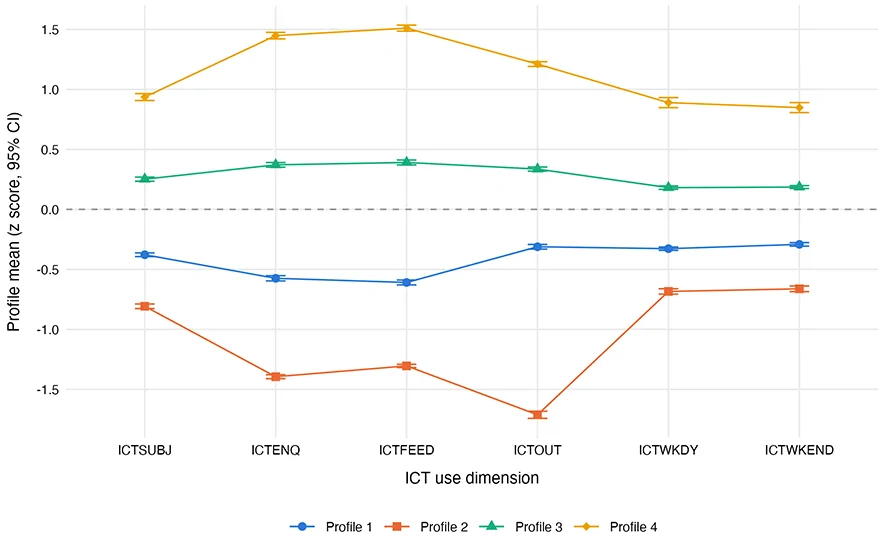
Profile means on the six ICT use dimensions (z scores).

*Profile 1: Below-average use* (33.2%; *n* = 28,056) was the second-largest profile. Its means lay 0.29 to 0.61 SD below the sample mean on all six dimensions, so these students used ICT somewhat less than average both for school and in their leisure time. Girls made up 55.9% of the profile, the highest share of any profile, and mean ESCS (−0.44) was similar to that of Profiles 3 and 4.

*Profile 2: Very low use* (15.0%; *n* = 8,463) had the lowest means on every dimension. The gap was largest for school-related use, above all for school activities outside the classroom (*z* = −1.71), enquiry-based learning (−1.39) and support or feedback via ICT (−1.30), and smaller for weekday and weekend leisure use (−0.68 and −0.66). This profile also stood out in its composition. Mean ESCS was −1.03, compared with −0.37 to −0.44 in the other profiles (SMD = 0.50 to 0.56; within countries 0.40 to 0.55), and girls made up only 42.4%. Profile 2 accordingly comprised 24.4% of students in the lowest ESCS quartile but only 7.5% of those in the highest.

*Profile 3: Above-average use* (39.0%; *n* = 36,571) was the largest profile. Its means were slightly above the sample mean on all dimensions (*z* = 0.18 to 0.39), and in ESCS (−0.37) and gender (50.3% girls) it differed little from Profile 4.

*Profile 4: Very high use* (12.8%; *n* = 10,573) had the highest means on all six dimensions, most of all for support or feedback via ICT (*z* = 1.51) and enquiry-based learning (1.45), and less so for weekday and weekend leisure use (0.89 and 0.85). Its share was nearly the same in every ESCS quartile (12.0% to 13.5%). Unexpectedly, students in this profile reported the lowest ICT availability, both outside school (ICTHOME −0.46 vs. −0.03 to −0.11; SMD = 0.30 to 0.37) and at school (ICTSCH −0.37 vs. −0.03 to −0.10; SMD = 0.23 to 0.28), and these differences remained when availability was centered within countries.

A key structural observation in Figure 3 is that the profiles differ mainly in level rather than in shape: their order is the same on all six dimensions. They are not fully parallel, however. The distance between the lowest and highest profile was about twice as large for enquiry-based learning, support or feedback via ICT and school activities outside the classroom (2.81 to 2.92 SD) as for weekday and weekend leisure use (1.57 and 1.51 SD); subject-related use in lessons lay in between (1.75 SD). The profiles therefore separate students more by how often they use ICT for school than by how much time they spend on it in their free time. Differences in immigrant background were small across all profiles (4.9% to 7.7%).

Profile shares varied considerably across countries; the share of Profile 4, for example, ranged from 2.2% in Germany to 33.2% in Albania (Supplementary Table S7). Because measurement invariance of the ICT use factors across countries was not tested, these differences are reported descriptively and are not interpreted.

### 4.3 Creative thinking across ICT use profiles (RQ3)

To examine whether creative thinking differed across the ICT use profiles, BCH-corrected mean scores were compared using all 10 plausible values, the final student weight and the 80 replicate weights (Section 3.5). The overall Wald test indicated a significant difference between the profiles, D1(3, 516.9) = 105.42, *p* < 0.001. Mean scores and pairwise comparisons are reported in Table 5 and Figure 4.

**Table 5:** Creative thinking scores by ICT use profile.

Profile/comparison	Pooled	Country fixed effects	Pooled + ESCS + gender	Country fixed effects + ESCS + gender
***A. Mean creative thinking score (SE)***				
Profile 1 (below-average use)	30.01 (0.23)	29.17 (0.20)	29.62 (0.19)	29.06 (0.18)
Profile 2 (very low use)	23.54 (0.33)	24.36 (0.28)	26.55 (0.28)	26.26 (0.27)
Profile 3 (above-average use)	28.06 (0.21)	27.90 (0.21)	27.42 (0.19)	27.54 (0.19)
Profile 4 (very high use)	24.41 (0.31)	26.12 (0.31)	24.27 (0.27)	25.68 (0.27)
***B. Pairwise differences in score points (d)***				
Profile 1 – Profile 2	6.47 (0.52)	4.81 (0.39)	3.07 (0.25)	2.81 (0.23)
Profile 1 – Profile 3	1.96 (0.16)	1.28 (0.10)	2.20 (0.18)	1.52 (0.12)
Profile 1 – Profile 4	5.60 (0.45)	3.05 (0.25)	5.35 (0.43)	3.39 (0.27)
Profile 2 – Profile 3	−4.52 (−0.36)	−3.53 (−0.28)	−0.87 (−0.07)^*^	−1.28 (−0.10)
Profile 2 – Profile 4	−0.87 (−0.07)^*^	−1.75 (−0.14)	2.28 (0.18)	0.58 (0.05)^*^
Profile 3 – Profile 4	3.65 (0.29)	1.78 (0.14)	3.14 (0.25)	1.86 (0.15)
Overall test, D1 (df)	105.42 (3, 516.9)	62.09 (3, 412.3)	—	—

BCH-corrected estimates weighted by the final student weight; all 10 plausible values were analyzed and combined with Rubin's rules, with standard errors from the 80 replicate weights. Pooled and country fixed effects models: *N* = 83,663 (42 countries). Models adjusted for ESCS and gender: students with complete covariates, *N* = 81,426 (41 countries; no ESCS data for Costa Rica). Adjusted means are evaluated at the weighted sample means of the covariates. *d* = difference divided by the pooled within-profile standard deviation (12.41). All differences are significant at *p* < 0.05 after Bonferroni correction for six comparisons except those marked ^*^. Overall tests (D1) compare all four profile means. Cohen's d: small ≥ 0.20, medium ≥ 0.50, large ≥ 0.80 (Cohen, 1988). Without covariates, the differences in the complete-covariate sample were 6.49 (Profile 1 – Profile 2) and 5.54 (Profile 1 – Profile 4).

**Figure 4:**
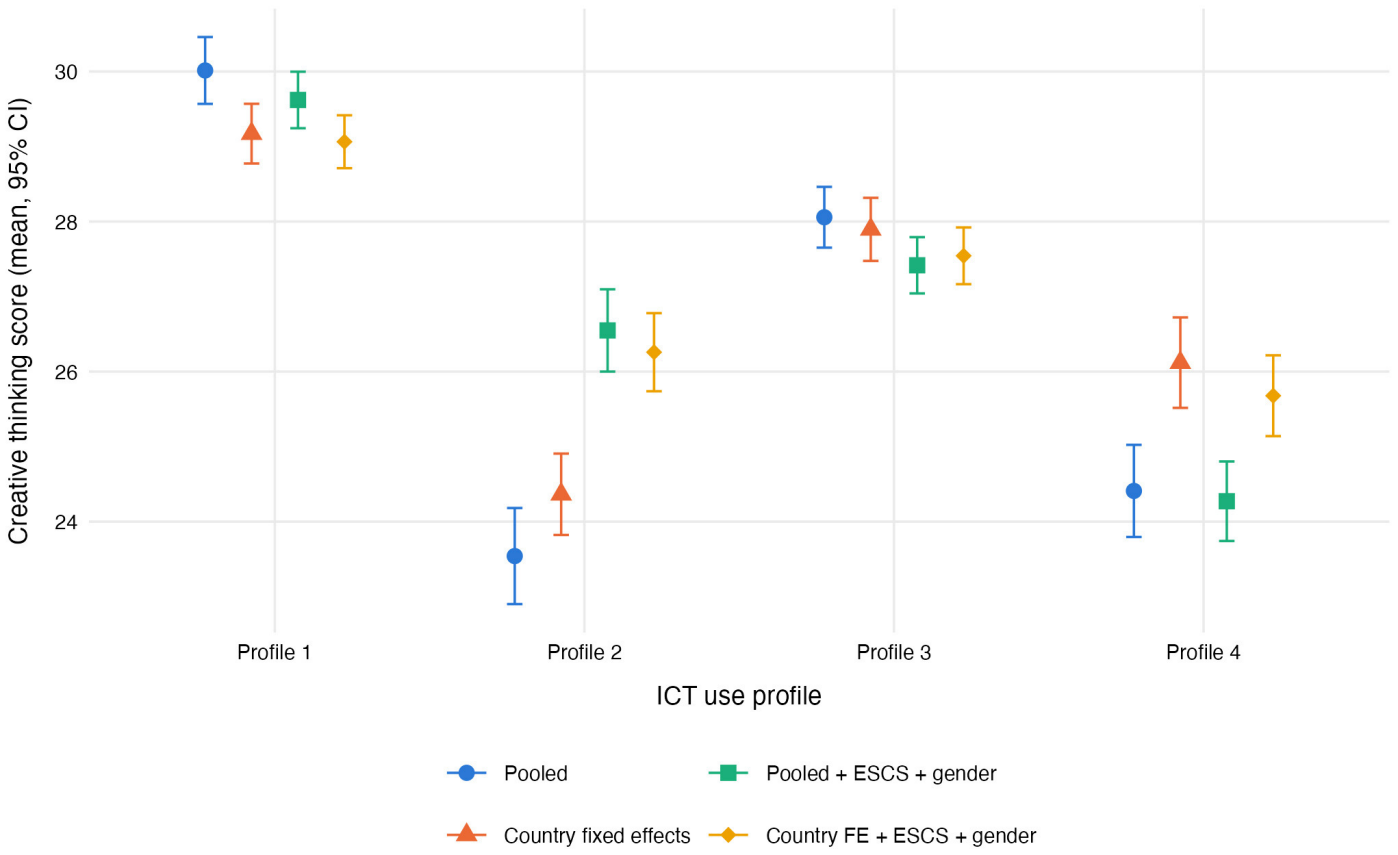
Estimated mean creative thinking scores by ICT use profile under four models.

Students in Profile 1 (below-average use) had the highest mean score (M = 30.01), followed by Profile 3 (above-average use; M = 28.06), whereas the two profiles at either end of the use spectrum, Profile 2 (very low use; M = 23.54) and Profile 4 (very high use; M = 24.41), scored lowest and did not differ from each other (difference = 0.87, *d* = 0.07, Bonferroni-adjusted *p* = 0.31). The largest differences were between Profile 1 and Profile 2 (6.47 points, *d* = 0.52, medium effect) and between Profile 1 and Profile 4 (5.60 points, *d* = 0.45, small to medium effect). With country fixed effects, the overall difference remained significant, D1(3, 412.3) = 62.09, *p* < 0.001. The differences were smaller but kept the same order (Profile 1: 29.17; Profile 3: 27.90; Profile 4: 26.12; Profile 2: 24.36), and all six pairwise differences were significant (*d* = 0.10 to 0.39).

Adjustment for ESCS and gender affected the two low-scoring profiles differently. In the sample with complete covariates (*n* = 81,426), the gap between Profile 1 and Profile 2 shrank from 6.49 to 3.07 points (*d* = 0.25), whereas the gap between Profile 1 and Profile 4 changed little (from 5.54 to 5.35 points, *d* = 0.43). With country fixed effects and both covariates, Profile 1 still scored highest (M = 29.06), and Profile 2 (M = 26.26) and Profile 4 (M = 25.68) did not differ (0.58 points, *p* = 0.85). The lower scores of students with very low use were thus partly associated with their socioeconomic background, whereas those of students with very high use were not.

These results held across subgroups and specifications. Profile 1 had the highest mean in every ESCS quartile and among both boys and girls, with and without country fixed effects, and its advantage over Profiles 2 and 4 was significant in all of these subgroups (*d* = 0.14 to 0.57; Supplementary Table S8). Among girls, Profile 4 scored higher than Profile 2 (3.16 points with country fixed effects), whereas among boys the two profiles did not differ. With senate weights, the order of the profiles was unchanged (31.65, 29.83, 25.39, and 24.55 for Profiles 1, 3, 4, and 2). The three-profile solution showed the same pattern: students with average use scored highest, and after adjustment for country, ESCS and gender the high-use profile scored lowest (Supplementary Table S6).

### 4.4 Relative importance of the ICT use profile in predicting creative thinking (RQ4)

To assess how the ICT use profile contributes to the prediction of creative thinking compared with the six ICT use dimensions from which it was derived, students' background and ICT availability, random forest models were estimated for nested sets of predictors (Section 3.5). Each set was evaluated by its weighted R^2^ in schools that had not been used to fit the model, and the contribution of a predictor group was taken as the change in R^2^ when it was added (Table 6). The analyses included 82,016 students in 41 countries; Costa Rica was excluded because ESCS was not available. At least one predictor value was imputed for 13,440 students (16.4%). These students scored markedly lower in creative thinking than students with complete predictor data (SMD = 0.74), and a larger share of them belonged to Profile 4 (21.2% vs. 10.3%).

**Table 6:** Prediction of creative thinking by random forest models.

Predictor set	Main analysis (imputed; *n* = 82,016)	Complete cases (*n* = 68,576)
***A. Weighted cross-validated R** ^2^ **, mean [min, max] over 10 plausible values***		
Profile	0.022 [0.020, 0.023]	0.024 [0.022, 0.025]
Six factor scores	0.165 [0.157, 0.170]	0.187 [0.181, 0.193]
Background + availability	0.261 [0.258, 0.267]	0.251 [0.247, 0.257]
Background + availability + profile	0.272 [0.267, 0.275]	0.261 [0.257, 0.265]
Background + availability + six factor scores	0.332 [0.328, 0.336]	0.334 [0.332, 0.341]
Background + availability + profile + six factor scores	0.332 [0.328, 0.337]	0.335 [0.333, 0.341]
Background + availability + posterior probabilities	0.262 [0.258, 0.266]	—
Background + availability + profile (random draw)	0.270 [0.266, 0.275]	—
Background + availability + country	0.360 [0.357, 0.367]	0.350 [0.346, 0.355]
Background + availability + profile + country	0.369 [0.365, 0.375]	0.360 [0.355, 0.363]
Background + availability + six factor scores + country	0.412 [0.406, 0.417]	0.412 [0.407, 0.416]
***B. Change in weighted cross-validated R** ^2^ **, mean [min, max] (plausible values with a gain)***		
Profile added to background + availability	0.0103 [0.0082, 0.0132] (10/10)	0.0102 [0.0080, 0.0131] (10/10)
Six factor scores added to background + availability	0.0704 [0.0624, 0.0778] (10/10)	0.0836 [0.0758, 0.0912] (10/10)
Profile added to background + availability + six factor scores	0.0006 [−0.0001, 0.0011] (9/10)	0.0009 [−0.0002, 0.0018] (9/10)
Six factor scores added to background + availability + profile	0.0608 [0.0550, 0.0655] (10/10)	0.0743 [0.0691, 0.0784] (10/10)
Posterior probabilities instead of modal profile	−0.0092 [−0.0111, −0.0067] (0/10)	—
Random profile draw instead of modal profile	−0.0014 [−0.0027, −0.0002] (0/10)	—
Profile added to background + availability + country	0.0084 [0.0061, 0.0105] (10/10)	0.0094 [0.0072, 0.0125] (10/10)
Six factor scores added to background + availability + country	0.0514 [0.0440, 0.0562] (10/10)	0.0616 [0.0541, 0.0665] (10/10)

R^2^ in held-out schools from five-fold cross-validation with schools assigned to folds within countries, weighted by the final student weight; each plausible value was analyzed with its own imputed dataset. Background = gender, ESCS, and immigrant background; availability = ICTHOME and ICTSCH; profile = modal profile of the four-profile solution; six factor scores = the ICT-use factor scores from which the profiles were derived. Costa Rica was excluded (no ESCS data). Unweighted R^2^, permutation importance, the item-level analysis and the three-profile solution are reported in Supplementary Table S9.

Profile membership alone explained 2.2% of the variance in creative thinking in held-out schools, and the six factor scores alone explained 16.5% (Table 6). Background and ICT availability reached *R*^2^ = 0.261. Adding the profile to these predictors raised R^2^ by 0.010 (in all 10 plausible values), whereas adding the six factor scores raised it by 0.070. Once the factor scores were included, the profile added little (Δ*R*^2^ = 0.0006, range −0.0001 to 0.0011). The same pattern held with country as an additional predictor: the factor scores added 0.051 and the profile 0.008 to a model with background, availability and country. Replacing the modal profile by the posterior probabilities lowered R^2^ by 0.009, and replacing it by a randomly drawn profile lowered it by 0.001, so the small contribution of the profile did not depend on how membership was represented.

Permutation importance showed the same picture. ESCS was by far the most important single predictor; permuting it increased the weighted prediction error by 47.5% in the model with background, availability and factor scores. The six factor scores together accounted for 28.5%, led by use for school activities outside the classroom (7.4%) and weekday leisure use (7.0%), followed by support or feedback via ICT (4.7%). Gender, immigrant background and ICT availability each accounted for less than 1.5%. In the model without factor scores, the profile ranked second after ESCS (3.4%), but when the factor scores were added its importance fell to 0.1% and was not positive in every plausible value.

In the complete-case analysis (*n* = 68,576), the factor scores again added 0.084 to background and availability, and the profile added only 0.0009 beyond them. In students who answered all 40 items (*n* = 47,059), the items predicted creative thinking somewhat better than the factor scores (Δ*R*^2^ = 0.052), but adding the profile to the items left R^2^ virtually unchanged (Δ*R*^2^ < 0.001; positive in 5 of 10 plausible values). With three profiles, the profile likewise added only 0.0006 beyond the factor scores (Supplementary Table S9).

Partial dependence plots show how the predicted score changed across the range of each predictor, holding the others at their observed values (Figure 5). For the profile, predictions followed the order of the ESCS- and gender-adjusted comparison in Section 4.3 (Profile 1: 28.3; Profile 3: 27.0; Profile 2: 25.8; Profile 4: 24.8; Supplementary Table S9). The factor scores showed different shapes. Predictions rose with use for school activities outside the classroom from 23.5 to about 27.8 and leveled off above the mean. For weekday leisure use, they were flat up to about the mean and then fell to 23.4, and weekend leisure use showed an inverted U. Predictions for support or feedback via ICT were highest at low levels (about 28.5) and fell to 24.4. Subject-related and enquiry-based use changed predictions by less than 2 points, and ICT availability by less than 1 point, whereas ESCS showed a steep, almost linear increase (from about 20 to 35). These curves describe model predictions, not effects.

**Figure 5:**
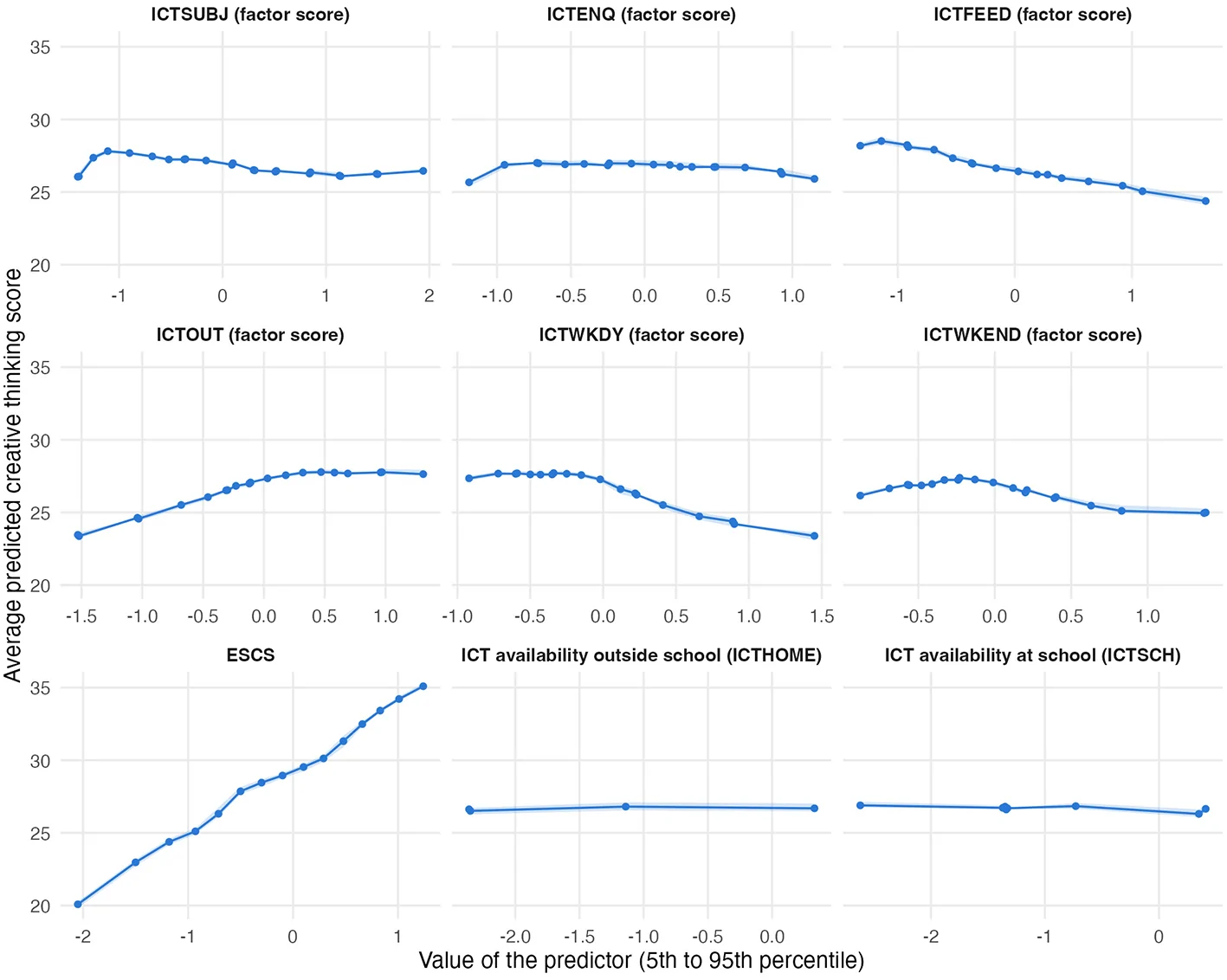
Partial dependence of predicted creative thinking on the ICT use factor scores, ESCS and ICT availability.

## 5 Discussion

This study examined how patterns of ICT use relate to creative thinking in PISA 2022. Students who used ICT somewhat below the average scored highest, followed by those somewhat above it, whereas those at either end of the use spectrum scored lowest. The low scores at the two ends, however, had different correlates: those of students with very low use were partly associated with their socioeconomic background, whereas those of students with very high use were not. In addition, profile membership contributed little to the prediction of creative thinking beyond the six ICT use dimensions from which the profiles were derived. The profiles diverged mainly in the overall level of ICT use, while the dimensions they summarize were related to creative thinking in different directions.

The ICT use pattern fits the idea that moderate use goes together with better outcomes than either very little or very much use. Gubbels et al. (2020) found a similar inverted U-shaped relationship between school-related ICT use and the reading scores of Dutch students in PISA 2015, and Przybylski and Weinstein (2017) showed that moderate digital screen use was not associated with lower wellbeing among English adolescents, with lower thresholds on weekdays than at weekends. A meta-analysis likewise showed that the association between digital device use and adolescents' academic performance depends on both purpose and amount: educational use was positively related to performance, whereas entertainment, social and gaming use, and longer use overall, were negatively related (Wang et al., 2024). Using PISA 2022 data, Cai et al. (2026) reported that ICT interaction behaviors predicted creative thinking in nonlinear and level-dependent ways rather than along a single slope. The present study adds to this work by examining the pattern in 42 countries and economies, using the six OECD dimensions of ICT use, and comparing students within countries and after adjustment for socioeconomic status and gender. Because Profile 1 used ICT less than average, the results also show that the highest scores did not depend on frequent use.

The two low-scoring profiles, however, appear to reflect different circumstances. Students with very low use came disproportionately from socioeconomically disadvantaged homes, and boys were overrepresented among them; the profile included about a quarter of students in the lowest ESCS quartile but fewer than one in 10 in the highest. Their deficit was largest for school-related use, especially schoolwork outside the classroom, rather than for leisure. Adjusting for ESCS and gender halved their gap to Profile 1. This is consistent with research on the digital usage gap, which shows that as access becomes widespread, differences in what people do online increasingly reflect existing social and educational inequalities (van Deursen and van Dijk, 2014). Very low use may therefore mark a broader disadvantage rather than being a reason for lower creative thinking in itself.

The gap for students with very high use followed a different pattern. It changed little after adjustment for ESCS and gender, and the share of this profile was nearly the same in every ESCS quartile. Its largest deviations were for support or feedback via ICT and enquiry-based learning, although its leisure use was also well above average. Very frequent use was thus associated with lower creative thinking regardless of socioeconomic background. A similar counterintuitive pattern has emerged from other analyses of the PISA 2022 creative thinking data. Kagan et al. (2026) reported a negative correlation between students' participation in creative extracurricular activities and their creative thinking performance, which persisted after controlling for psychosocial and contextual factors. One interpretation is that how often students do something says little about the quality of thinking it involves. Kraus et al. (2026) also found that academic competences in mathematics, reading and science were among the strongest predictors of item scores on the creative thinking assessment, whereas self-reported creativity and creative activities were of limited relevance. Students who struggle academically may therefore score lower on the assessment regardless of how they use ICT.

Several explanations are possible for the lower scores of students with very high use, and the present data cannot distinguish between them. First, the association may run in the opposite direction: students who struggle at school may receive more digital support and feedback, so that frequent school-related use reflects lower achievement rather than leading to it. This possibility is taken up again below for the feedback dimension. Second, very frequent use may take time away from other activities (Przybylski and Weinstein, 2017). Third, part of the profile may reflect a tendency to choose the upper end of response scales. Students in Profile 4 reported the highest use on every dimension but the lowest ICT availability, which is consistent with an extreme response style, and because such response styles vary mostly within countries, country fixed effects would not remove them (Lu and Bolt, 2015). Response style, however, is unlikely to explain the lower creative thinking scores themselves. The association between extreme response style and achievement is negative but small (*d* = −0.04 in Dibek and Yildirim-Erbasli, 2026; *r* = −0.02 in Lu and Bolt, 2015), whereas the adjusted gap between Profiles 1 and 4 was *d* = 0.43.

In the random forest models, the six ICT use dimensions predicted creative thinking better than profile membership did. Adding the profile to a model with the dimensions, background and ICT availability changed the explained variance by less than a tenth of a percentage point, whereas adding the dimensions to a model with the profile raised it by about six percentage points. One reason is that the profiles differed mainly in the overall level of use, as profiles of ICT use in earlier PISA cycles have also done (Karakoyun and Başaran, 2022; Kim and Kim, 2023; Xiao and Sun, 2022). Because the profiles were ordered in the same way on all six dimensions, profile membership mainly indicates how much a student uses ICT overall. It cannot separate use for purposes that are positively related to creative thinking from use for purposes that are negatively related to it, so part of the information carried by the dimensions is lost. In a systematic review of PISA studies, Kim and Liou (2026) concluded that the association between students' ICT use and achievement depends on the educational setting, such as the subject and the instructional design; the present results suggest that such differences are easily lost when use is summarized mainly by its level. That the models explained only a modest share of variance is not surprising. ESCS was by far the most important predictor, in line with Goecke et al. (2026), who found across 62 countries that socioeconomic status was a strong predictor of creative thinking, as it was of mathematics and reading. Fu et al. (2026) also found that creative personality predicted creative thinking, and that a supportive creative environment at home and among peers weakened this link. The measurement challenges of the PISA 2022 creative thinking assessment noted by several authors may also limit how much variance any set of predictors can explain (Barbot and Kaufman, 2025; Kraus et al., 2026; Rutkowski and Rutkowski, 2025). It should also be noted that permutation importance shows how much a predictor contributes to prediction, not the direction or cause of its association.

The partial dependence plots suggest why the profiles carried less information than the dimensions. Predicted scores rose with school activities outside the classroom up to about the mean and then leveled off, fell when weekday leisure use exceeded the mean, followed an inverted U for weekend leisure use, and were lower at higher levels of support or feedback via ICT. Melnikova et al. (2026) found a similar set of directions for mathematics, reading, and science among Lithuanian students in PISA 2022: school-related use outside the classroom was positively associated with achievement, whereas feedback via ICT and leisure use were negatively associated. High weekday leisure use was associated with a steeper decline than high weekend use, and Przybylski and Weinstein (2017) likewise found that the association between screen time and wellbeing differed between weekdays and weekends. The negative association for feedback may be the clearest case of reverse direction, because support and feedback tend to go to students who need them (Melnikova et al., 2026; see also Hu and Wang, 2022, for teacher feedback and reading), although digital feedback from teachers, as distinct from other sources, has been linked to better self-regulated learning and mathematics achievement (Xiong et al., 2026). Because Profile 4 deviated most strongly on this dimension, the same mechanism may partly explain its low scores. ICT availability at home and at school, in contrast, was almost unrelated to predicted creative thinking once background and use were included, consistent with Tian J. et al. (2026), who found that the positive association between ICT availability at home and academic achievement diminished or turned negative once family and school socioeconomic status were controlled. More broadly, studies of earlier and current PISA cycles have found that the quality and purpose of ICT use relate to achievement more closely than its frequency (Hristov et al., 2025; Petko et al., 2017).

For practice, the findings suggest that the frequency of ICT use is a poor guide on its own, although cross-sectional data cannot show whether changing students' use would change their creative thinking. Students with very low use came disproportionately from disadvantaged homes and used ICT least for school, so supporting them may require attention to how technology is used for learning, not only to access (Kim and Liou, 2026). At the other end, frequent digital support and feedback may signal that a student is struggling rather than that technology is being used productively. Neither result supports simple recommendations to reduce or increase screen time.

Methodologically, the study shows both the value and the limits of person-centered profiles in ICT research (Spurk et al., 2020). The profiles made it possible to describe who the students with very low and very high use were and to show that the two ends of the use spectrum differ in socioeconomic composition. For prediction, however, the dimensions from which the profiles were built were more informative. Studies using PISA and similar data may therefore benefit from reporting person-centered profiles together with variable-centered models of the underlying dimensions, especially when these dimensions may relate to the outcome in different directions.

## 6 Limitations and future directions

Several limitations should be considered when interpreting these findings. Because the PISA 2022 data are cross-sectional, the direction of the relationship between ICT use and creative thinking cannot be determined, and both may depend on other factors. The comparisons were adjusted for country, ESCS and gender, but not for prior achievement, motivation or school context. These factors may be related to both profile membership and creative thinking; academic competences are closely related to creative thinking scores (Kraus et al., 2026). All ICT use measures were self-reported and describe how often students used ICT, not what they did with it or how well. Self-reports may be affected by recall error and by response styles such as extreme responding (Lu and Bolt, 2015), and Profile 4 may partly reflect such a style.

The findings refer only to the 42 countries and economies that administered the optional ICT questionnaire, whose students scored higher than test-takers in the other 21 countries. Within these countries, students who answered none of the ICT items had lower ESCS and lower creative thinking scores than those included. In the random forest models, the 16.4% of students with at least one imputed predictor scored markedly lower than the others; the complete-case analysis led to the same conclusions, but imputed values may still be less accurate for these students. Measurement invariance of the ICT use factors across countries was not tested, so profile shares were not compared between countries, and country fixed effects remove only differences in mean levels.

The profile labels summarize the level of use and were chosen by the authors; other labels could be defended. The indicators covered the six OECD dimensions of ICT use but not ICT use in mathematics lessons (IC184), which is not part of these dimensions. Finally, three of the school-related dimensions were strongly correlated (factor scores, *r* = 0.65), so parts of the partial dependence curves may rest on combinations of values that are rare in the data, and these curves describe the fitted models rather than causal relationships.

Future research could address these limitations in several ways. Longitudinal or experimental designs would show whether changes in ICT use precede or follow changes in creative thinking, and data on prior achievement and motivation would help separate ICT use from general academic performance. Studies that record what students actually do with technology, for example through log data or interviews, could show whether the negative association for digital feedback reflects support given to struggling students. Testing the measurement invariance of the ICT use dimensions would allow profile shares and associations to be compared across countries. Finally, analyses of the four creative thinking domains (written expression, visual expression, scientific problem-solving, and social problem-solving) could show whether the associations differ by domain, a question the present analysis of the overall score could not address.

## 7 Conclusion

Using PISA 2022 data from 42 countries and economies, this study compared creative thinking across profiles of students' ICT use. Four profiles emerged from LPA, of which those with below-average use scored highest, followed by those with above-average use, and those with very low and very high use scored lowest.

The two low-scoring groups differed in an important way. The lower scores of students with very low use were partly associated with socioeconomic disadvantage, whereas those of students with very high use were not. Profile membership also predicted creative thinking less well than the six ICT use dimensions from which it was derived, partly because these dimensions were related to creative thinking in different directions.

For research on ICT and creative thinking, the main implication is that neither the amount of use nor a single profile of use is enough to describe this relationship. Studies need to consider both how much and for what purposes students use ICT, and to take their socioeconomic background into account. Longitudinal data will be needed to show whether the patterns found here reflect the consequences of ICT use, the support given to students who are struggling, or both.

## Data Availability

Publicly available datasets were analyzed in this study. This data can be found here: https://www.oecd.org/pisa/data/2022database/.
