# Supplementary material for: Adolescent ICT use profiles and creative thinking: evidence from 42 countries in PISA 2022
Source: Front Psychol. 2026 Oct 29;17:1950010. doi: 10.3389/fpsyg.2026.1950010 (PMC13754889; doi:10.3389/fpsyg.2026.1950010)
Supplement: Supplementary file 1 [file Supplementary_File_1.docx]

**Supplementary**

**Supplementary Table S1.** Sample flow by country (42 countries and economies in the analysis sample)

| **Country** | **Student file** | **Computer-based** | **Creative thinking form** | **Test-takers** | **In ICT country** | **Analysis sample** | **% of test-takers** |
| --- | --- | --- | --- | --- | --- | --- | --- |
| ALB | 6,129 | 6,129 | 1,674 | 1,600 | 1,600 | 1,258 | 78.6 |
| AUS | 13,437 | 13,437 | 3,776 | 3,703 | 3,703 | 3,418 | 92.3 |
| BEL | 8,286 | 8,286 | 2,396 | 2,388 | 2,388 | 2,175 | 91.1 |
| BGR | 6,107 | 6,107 | 1,929 | 1,890 | 1,890 | 1,582 | 83.7 |
| BRA | 10,798 | 10,798 | 3,390 | 3,352 | 3,352 | 2,654 | 79.2 |
| BRN | 5,576 | 5,576 | 1,568 | 1,560 | 1,560 | 1,524 | 97.7 |
| CHL | 6,488 | 6,488 | 1,692 | 1,672 | 1,672 | 1,392 | 83.3 |
| CRI | 6,113 | 6,113 | 1,741 | 1,726 | 1,726 | 1,647 | 95.4 |
| CZE | 8,460 | 8,460 | 2,635 | 2,630 | 2,630 | 2,475 | 94.1 |
| DEU | 6,116 | 6,116 | 1,697 | 1,685 | 1,685 | 1,453 | 86.2 |
| DNK | 6,200 | 6,200 | 1,877 | 1,865 | 1,865 | 1,679 | 90.0 |
| DOM | 6,868 | 6,868 | 1,955 | 1,947 | 1,947 | 1,703 | 87.5 |
| ESP | 30,800 | 30,800 | 8,833 | 8,799 | 8,799 | 8,256 | 93.8 |
| EST | 6,392 | 6,392 | 1,775 | 1,765 | 1,765 | 1,641 | 93.0 |
| FIN | 10,239 | 10,239 | 2,877 | 2,851 | 2,851 | 2,533 | 88.8 |
| GRC | 6,403 | 6,403 | 1,781 | 1,765 | 1,765 | 1,680 | 95.2 |
| HKG | 5,907 | 5,907 | 1,663 | 1,645 | 1,645 | 1,542 | 93.7 |
| HRV | 6,135 | 6,135 | 1,710 | 1,699 | 1,699 | 1,618 | 95.2 |
| HUN | 6,198 | 6,198 | 1,932 | 1,930 | 1,930 | 1,847 | 95.7 |
| ISL | 3,360 | 3,360 | 907 | 893 | 893 | 806 | 90.3 |
| ISR | 6,251 | 6,251 | 1,733 | 1,702 | 1,702 | 1,302 | 76.5 |
| ITA | 10,552 | 10,552 | 3,322 | 3,311 | 3,311 | 3,158 | 95.4 |
| JOR | 7,799 | 7,799 | 2,175 | 2,167 | 2,167 | 1,932 | 89.2 |
| KAZ | 19,769 | 19,769 | 5,535 | 5,521 | 5,521 | 5,373 | 97.3 |
| KOR | 6,454 | 6,454 | 1,812 | 1,796 | 1,796 | 1,710 | 95.2 |
| LTU | 7,257 | 7,257 | 2,004 | 1,985 | 1,985 | 1,875 | 94.5 |
| LVA | 5,373 | 5,373 | 1,527 | 1,522 | 1,522 | 1,430 | 94.0 |
| MAC | 4,384 | 4,384 | 1,231 | 1,230 | 1,230 | 1,230 | 100.0 |
| MAR | 6,867 | 6,867 | 1,911 | 1,897 | 1,897 | 1,797 | 94.7 |
| MLT | 3,127 | 3,127 | 875 | 866 | 866 | 785 | 90.6 |
| MYS | 7,069 | 7,069 | 2,223 | 2,216 | 2,216 | 2,141 | 96.6 |
| PAN | 4,544 | 4,544 | 1,280 | 1,257 | 1,257 | 1,097 | 87.3 |
| POL | 6,011 | 6,011 | 1,911 | 1,904 | 1,904 | 1,773 | 93.1 |
| QUR | 3,876 | 3,876 | 1,110 | 1,081 | 1,081 | 932 | 86.2 |
| ROU | 7,364 | 7,364 | 2,061 | 2,040 | 2,040 | 1,976 | 96.9 |
| SAU | 6,928 | 6,928 | 2,175 | 2,167 | 2,167 | 1,898 | 87.6 |
| SGP | 6,606 | 6,606 | 1,846 | 1,838 | 1,838 | 1,807 | 98.3 |
| SVK | 5,824 | 5,824 | 1,607 | 1,595 | 1,595 | 1,502 | 94.2 |
| SVN | 6,721 | 6,721 | 1,822 | 1,812 | 1,812 | 1,748 | 96.5 |
| TAP | 5,857 | 5,857 | 1,610 | 1,598 | 1,598 | 1,563 | 97.8 |
| THA | 8,495 | 8,495 | 2,359 | 2,352 | 2,352 | 2,301 | 97.8 |
| URY | 6,618 | 6,618 | 1,849 | 1,834 | 1,834 | 1,450 | 79.1 |
| **Total** | **319,758** | **319,758** | **91,786** | **91,056** | **91,056** | **83,663** | **91.9** |

*Note.* Countries are listed by their three-letter PISA codes. Test-takers = students with at least one scored creative thinking response. Analysis sample = test-takers who answered at least one of the 40 ICT-use items. Excluded countries (not shown): ARE, ARG, AUT, CAN, CHE, COL, FRA, GBR, GEO, GTM, IDN, IRL, JAM, JPN, KHM, KSV, MDA, MEX, MKD, MNE, MNG, NLD, NOR, NZL, PER, PHL, PRT, PRY, PSE, QAT, QAZ, SLV, SRB, SWE, TUR, USA, UZB, VNM (paper-based administration, no creative thinking assessment, or no ICT questionnaire; see Table 1).

**Supplementary Table S2.** Variables used in the study

| **Variable** | **PISA 2022 code** | **Description** | **Response scale / coding** | **Use in the analyses** |
| --- | --- | --- | --- | --- |
| *Outcome* | | | | |
| Creative thinking | PV1CRTH_NC–PV10CRTH_NC | Ten plausible values of creative thinking | 0–60 score scale | Outcome in profile comparisons and random forest models; each PV analysed separately |
| *ICT use: 40 items of the ICT familiarity questionnaire (indicators of the confirmatory factor analysis)* | | | | |
| *ICTSUBJ: Subject-related ICT use during lessons (IC173, 4 items)* | | | | |
| How often use [digital resources] in lessons in: | IC173Q01JA | [Test language] | 5-point frequency scale; code 6 ("I do not have this subject") set to missing | Indicator of ICTSUBJ (CFA) |
|  | IC173Q02JA | [Mathematics] |  |  |
|  | IC173Q03JA | [Science] |  |  |
|  | IC173Q04JA | [Computer science], [information technology], [informatics] or similar lessons. |  |  |
| *ICTENQ: Use of ICT in enquiry-based learning activities (IC174, 10 items)* | | | | |
| How often used [digital resources] to: | IC174Q01JA | Create a multi-media presentation with pictures, sound or video | 5-point frequency scale | Indicator of ICTENQ (CFA) |
|  | IC174Q02JA | Write or edit text for a school assignment (e.g. using [Google® Docs™], [Microsoft® Word]) |  |  |
|  | IC174Q03JA | Find information online about real-world problems or phenomena (e.g. climate change, oil spills, measuring the height of a building) |  |  |
|  | IC174Q04JA | Collect and record data (e.g. using data loggers, [Microsoft® Access™], [Google® Forms], spreadsheets) |  |  |
|  | IC174Q05JA | Analyse data that you have collected yourself (e.g. using [Microsoft® Excel™]) |  |  |
|  | IC174Q06JA | Report or share your results from your own experiments or investigations |  |  |
|  | IC174Q07JA | Plan and manage work or projects (e.g. dividing tasks, managing deadlines) |  |  |
|  | IC174Q08JA | Track the progress of your own work projects |  |  |
|  | IC174Q09JA | Collaborate with other students to create digital content (e.g. presentation, etc.) |  |  |
|  | IC174Q10JA | Play digital learning games (e.g. [Atlantis Remixed], [Duolingo®]) |  |  |
| *ICTFEED: Support or feedback via ICT (IC175, 4 items)* | | | | |
| How often used [digital resources] to: | IC175Q01JA | Read or listen to feedback sent by my teachers regarding my work and academic results | 5-point frequency scale | Indicator of ICTFEED (CFA) |
|  | IC175Q02JA | Read or listen to feedback sent by other students on my work |  |  |
|  | IC175Q03JA | Read or listen to feedback automatically selected for me by an educational software or app |  |  |
|  | IC175Q05JA | Work on drill and practice exercises using an educational software or app |  |  |
| *ICTOUT: Use of ICT for school activities outside the classroom (IC176, 8 items)* | | | | |
| How often used [digital resources] to: | IC176Q01JA | See my grades or results from specific assignments (e.g. homework or tests) | 5-point frequency scale | Indicator of ICTOUT (CFA) |
|  | IC176Q02JA | Browse the Internet for schoolwork (e.g. for preparing an essay or presentation) |  |  |
|  | IC176Q03JA | Browse the Internet to follow up lessons (e.g. for finding explanations) |  |  |
|  | IC176Q04JA | Receive or download assignments or instructions (via [digital resources]) from my teacher |  |  |
|  | IC176Q05JA | Upload my work (e.g. documents or presentations) to a school portal or another platform to share materials with my teacher and/or students |  |  |
|  | IC176Q06JA | Communicate with my teacher |  |  |
|  | IC176Q07JA | Communicate with other students about schoolwork |  |  |
|  | IC176Q08JA | Search for information on school-related activities or assignments |  |  |
| *ICTWKDY: Leisure ICT use on a typical weekday (IC177, 7 items)* | | | | |
| Weekday. How much time spent: | IC177Q01JA | Play video-games (using my smartphone, a gaming console or an online platform or apps) | 6-point time scale (1 = no time at all, 6 = more than 7 hours) | Indicator of ICTWKDY (CFA) |
|  | IC177Q02JA | Browse social networks (e.g. [Instagram®], [Facebook®]) |  |  |
|  | IC177Q03JA | Browse the Internet (excluding social networks) for fun (e.g. reading news, listening to podcasts and music or watching videos) |  |  |
|  | IC177Q04JA | Look for practical information online (e.g. find a place, book a train ticket, buy a product) |  |  |
|  | IC177Q05JA | Communicate and share digital content on social networks or any communication platform (e.g. [Facebook®], [Instagram®], [Twitter®], emails, chat) |  |  |
|  | IC177Q06JA | Read, listen to or view informational materials to learn how to do something (e.g. tutorial, podcast) |  |  |
|  | IC177Q07JA | Create or edit my own digital content (pictures, videos, music, computer programs) |  |  |
| *ICTWKEND: Leisure ICT use on a typical weekend day (IC178, 7 items)* | | | | |
| Weekend day. How much time using [digital resources] to: | IC178Q01JA | Play video-games (using my smartphone, a gaming console or an online platform or apps) | 6-point time scale (1 = no time at all, 6 = more than 7 hours) | Indicator of ICTWKEND (CFA) |
|  | IC178Q02JA | Browse social networks (e.g. Instagram®, Facebook®) |  |  |
|  | IC178Q03JA | Browse the Internet (excluding social networks) for fun (e.g. reading news, listening to podcasts and music or watching videos) |  |  |
|  | IC178Q04JA | Look for practical information online (e.g. find a place, book a train ticket, buy a product) |  |  |
|  | IC178Q05JA | Communicate and share digital content on social networks or any communication platform (e.g. Facebook®, Instagram®, Twitter®, emails, chat) |  |  |
|  | IC178Q06JA | Read, listen to or view informational materials to learn how to do something (e.g. tutorial, podcast) |  |  |
|  | IC178Q07JA | Create or edit my own digital content (pictures, videos, music, computer programs) |  |  |
| *Indicators derived in this study* | | | | |
| ICT-use factor scores | ICTSUBJ, ICTENQ, ICTFEED, ICTOUT, ICTWKDY, ICTWKEND | Factor scores from the six-factor CFA (Mplus, MLR/FIML); set to missing when no item of the factor was answered | Continuous | LPA indicators; random forest predictors |
| ICT-use profile | — | Most likely profile (modal assignment) and posterior probabilities from the LPA (K = 4) | Categorical (4 profiles); probabilities 0–1 | BCH comparisons; random forest predictors |
| *Background and ICT availability* | | | | |
| Gender | ST004D01T | Student gender | Girl = 1, boy = 0 | Adjusted and subgroup comparisons; random forest predictor |
| Socioeconomic status | ESCS | Index of economic, social and cultural status: composite of parents' highest education (PAREDINT), highest occupational status (HISEI) and home possessions (HOMEPOS) (OECD, 2023b) | Continuous; 0 = OECD average, 1 = OECD SD | Adjusted comparison; ESCS quartiles; random forest predictor |
| Immigrant background | IMMIG | OECD index based on the country of birth of the student and parents (ST019): 1 = native, 2 = second generation, 3 = first generation | Recoded: second or first generation = 1, native = 0; missing if the student or both parents did not respond | Profile composition; random forest predictor; categories 1–3 for profile shares by group |
| ICT availability outside school | ICTHOME | Availability of ICT outside school | Continuous (OECD index) | Profile description; random forest predictor |
| ICT availability at school | ICTSCH | Availability of ICT at school | Continuous (OECD index) | Profile description; random forest predictor |
| *Weights and design variables* | | | | |
| Final student weight | W_FSTUWT | Final student weight | — | All weighted estimates; case weights in random forests |
| Replicate weights | W_FSTURWT1–W_FSTURWT80 | Balanced repeated replication weights | Fay adjustment k = 0.5 | Standard errors |
| Senate weight | SENWT | Senate weight | Rescaled to 5,000 per country | Sensitivity analyses (CFA, profile comparisons) |
| Country | CNT | Country or economy | 42 categories (41 in the random forest models; Costa Rica lacks ESCS) | Country fixed effects; random forest predictor; fold assignment |
| School | CNTSCHID | School identifier | — | Clustering (CFA sensitivity); school-grouped cross-validation folds |
| Stratum | STRATUM | Explicit sampling stratum | — | Stratification (CFA sensitivity) |

*Note.* Item wording follows the PISA 2022 ICT familiarity questionnaire; bracketed terms are adapted nationally. PISA missing-data codes (valid skip, not applicable, invalid, no response) were set to missing for all variables. The factor scores were estimated in this study and are not the OECD indices of the same name in the PISA database. CFA = confirmatory factor analysis; LPA = latent profile analysis; MLR = robust maximum likelihood; FIML = full information maximum likelihood; BCH = Bolck–Croon–Hagenaars method; PV = plausible value.

**Supplementary Table S3.** Missing responses to the 40 ICT-use items in the analysis sample (N = 83,663)

| **Item** | **Label (PISA 2022 codebook)** | **Answered** | **Code 6 set to missing** | **No response / other missing** | **% missing** |
| --- | --- | --- | --- | --- | --- |
| IC173Q01JA | [Test language] | 79,036 | 1,230 | 3,397 | 5.5 |
| IC173Q02JA | [Mathematics] | 79,033 | 1,016 | 3,614 | 5.5 |
| IC173Q03JA | [Science] | 77,069 | 2,913 | 3,681 | 7.9 |
| IC173Q04JA | [Computer science], [information technology], [informatics] or similar lessons. | 65,497 | 14,537 | 3,629 | 21.7 |
| IC174Q01JA | Create a multi-media presentation with pictures, sound or video | 77,716 | — | 5,947 | 7.1 |
| IC174Q02JA | Write or edit text for a school assignment (e.g. using [Google® Docs™], [Microsoft® Word]) | 77,224 | — | 6,439 | 7.7 |
| IC174Q03JA | Find information online about real-world problems or phenomena (e.g. climate change, oil spills, measuring the height of a building) | 77,285 | — | 6,378 | 7.6 |
| IC174Q04JA | Collect and record data (e.g. using data loggers, [Microsoft® Access™], [Google® Forms], spreadsheets) | 77,077 | — | 6,586 | 7.9 |
| IC174Q05JA | Analyse data that you have collected yourself (e.g. using [Microsoft® Excel™]) | 76,724 | — | 6,939 | 8.3 |
| IC174Q06JA | Report or share your results from your own experiments or investigations | 76,629 | — | 7,034 | 8.4 |
| IC174Q07JA | Plan and manage work or projects (e.g. dividing tasks, managing deadlines) | 76,992 | — | 6,671 | 8.0 |
| IC174Q08JA | Track the progress of your own work projects | 76,542 | — | 7,121 | 8.5 |
| IC174Q09JA | Collaborate with other students to create digital content (e.g. presentation, etc.) | 76,970 | — | 6,693 | 8.0 |
| IC174Q10JA | Play digital learning games (e.g. [Atlantis Remixed], [Duolingo®]) | 76,718 | — | 6,945 | 8.3 |
| IC175Q01JA | Read or listen to feedback sent by my teachers regarding my work and academic results | 78,311 | — | 5,352 | 6.4 |
| IC175Q02JA | Read or listen to feedback sent by other students on my work | 77,876 | — | 5,787 | 6.9 |
| IC175Q03JA | Read or listen to feedback automatically selected for me by an educational software or app | 77,847 | — | 5,816 | 7.0 |
| IC175Q05JA | Work on drill and practice exercises using an educational software or app | 77,784 | — | 5,879 | 7.0 |
| IC176Q01JA | See my grades or results from specific assignments (e.g. homework or tests) | 77,202 | — | 6,461 | 7.7 |
| IC176Q02JA | Browse the Internet for schoolwork (e.g. for preparing an essay or presentation) | 76,628 | — | 7,035 | 8.4 |
| IC176Q03JA | Browse the Internet to follow up lessons (e.g. for finding explanations) | 76,389 | — | 7,274 | 8.7 |
| IC176Q04JA | Receive or download assignments or instructions (via [digital resources]) from my teacher | 76,410 | — | 7,253 | 8.7 |
| IC176Q05JA | Upload my work (e.g. documents or presentations) to a school portal or another platform to share materials with my teacher and/or students | 76,724 | — | 6,939 | 8.3 |
| IC176Q06JA | Communicate with my teacher | 76,421 | — | 7,242 | 8.7 |
| IC176Q07JA | Communicate with other students about schoolwork | 76,726 | — | 6,937 | 8.3 |
| IC176Q08JA | Search for information on school-related activities or assignments | 76,572 | — | 7,091 | 8.5 |
| IC177Q01JA | Play video-games (using my smartphone, a gaming console or an online platform or apps) | 76,981 | — | 6,682 | 8.0 |
| IC177Q02JA | Browse social networks (e.g. [Instagram®], [Facebook®]) | 76,259 | — | 7,404 | 8.8 |
| IC177Q03JA | Browse the Internet (excluding social networks) for fun (e.g. reading news, listening to podcasts and music or watching videos) | 76,683 | — | 6,980 | 8.3 |
| IC177Q04JA | Look for practical information online (e.g. find a place, book a train ticket, buy a product) | 76,216 | — | 7,447 | 8.9 |
| IC177Q05JA | Communicate and share digital content on social networks or any communication platform (e.g. [Facebook®], [Instagram®], [Twitter®], emails, chat) | 76,677 | — | 6,986 | 8.4 |
| IC177Q06JA | Read, listen to or view informational materials to learn how to do something (e.g. tutorial, podcast) | 76,512 | — | 7,151 | 8.5 |
| IC177Q07JA | Create or edit my own digital content (pictures, videos, music, computer programs) | 76,463 | — | 7,200 | 8.6 |
| IC178Q01JA | Play video-games (using my smartphone, a gaming console or an online platform or apps) | 74,887 | — | 8,776 | 10.5 |
| IC178Q02JA | Browse social networks (e.g. Instagram®, Facebook®) | 74,195 | — | 9,468 | 11.3 |
| IC178Q03JA | Browse the Internet (excluding social networks) for fun (e.g. reading news, listening to podcasts and music or watching videos) | 74,526 | — | 9,137 | 10.9 |
| IC178Q04JA | Look for practical information online (e.g. find a place, book a train ticket, buy a product) | 74,244 | — | 9,419 | 11.3 |
| IC178Q05JA | Communicate and share digital content on social networks or any communication platform (e.g. Facebook®, Instagram®, Twitter®, emails, chat) | 74,608 | — | 9,055 | 10.8 |
| IC178Q06JA | Read, listen to or view informational materials to learn how to do something (e.g. tutorial, podcast) | 74,393 | — | 9,270 | 11.1 |
| IC178Q07JA | Create or edit my own digital content (pictures, videos, music, computer programs) | 74,310 | — | 9,353 | 11.2 |

*Missing responses by block*

| **Block** | **Items** | **All items answered, %** | **Some items answered, %** | **No item answered, %** |
| --- | --- | --- | --- | --- |
| IC173 | 4 | 74.7 | 21.6 | 3.6 |
| IC174 | 10 | 85.5 | 10.4 | 4.1 |
| IC175 | 4 | 91.6 | 2.9 | 5.5 |
| IC176 | 8 | 86.6 | 8.2 | 5.2 |
| IC177 | 7 | 87.5 | 6.7 | 5.8 |
| IC178 | 7 | 85.5 | 5.6 | 9.0 |

*Note.* Code 6 in IC173 ("I do not have this subject") does not express a frequency and was set to missing; in IC177 and IC178 code 6 is the highest valid category. 48,031 students (57.4%) answered all 40 items. Missing item responses were handled by full information maximum likelihood.

**Supplementary Table S4.** Fit of latent profile models with one to eight profiles: local independence model (N = 83,663)

| **K** | **LL** | **Par.** | **BIC** | **CAIC** | **AWE** | **ICL-BIC** | **Entropy** | **Smallest (%)** | **Min. AvePP** | **Min. OCC** | **VLMR p** | **LMR p** | **ΔBIC (main − this model)** |
| --- | --- | --- | --- | --- | --- | --- | --- | --- | --- | --- | --- | --- | --- |
| 1 | −559,650.6 | 12 | 1,119,437 | 1,119,449 | 1,119,609 | 1,119,437 | — | 100.0 | — | — | — | — | −110,022 |
| 2 | −508,464.4 | 19 | 1,017,144 | 1,017,163 | 1,017,416 | 1,045,947 | .751 | 49.7 | .925 | 12.2 | .333 | .333 | −96,197 |
| 3 | −484,138.7 | 26 | 968,572 | 968,598 | 968,945 | 1,010,415 | .772 | 18.3 | .873 | 8.1 | < .001 | < .001 | −80,512 |
| 4 | −469,877.3 | 33 | 940,129 | 940,162 | 940,602 | 987,916 | .794 | 8.1 | .852 | 11.0 | < .001 | < .001 | −64,616 |
| 5 | −458,570.2 | 40 | 917,594 | 917,634 | 918,167 | 974,269 | .789 | 7.5 | .834 | 9.1 | < .001 | < .001 | −47,870 |
| 6 | −452,152.4 | 47 | 904,837 | 904,884 | 905,511 | 961,882 | .809 | 2.8 | .829 | 9.9 | < .001 | < .001 | −42,232 |
| 7 | −446,626.1 | 54 | 893,864 | 893,918 | 894,638 | 956,769 | .806 | 2.3 | .790 | 10.1 | < .001 | < .001 | −36,875 |
| 8 | −442,445.9 | 61 | 885,583 | 885,644 | 886,458 | 959,847 | .786 | 2.2 | .790 | 10.5 | < .001 | < .001 | −34,237 |

*Note.* Local independence model: profile-specific means, variances equal across profiles, no within-profile covariances. ΔBIC = BIC of the main model (weekday–weekend covariance; Table 3) minus BIC of the local independence model with the same number of profiles; negative values favour the main model. Abbreviations as in Table 3.

**Supplementary Table S5.** Profile sizes and profile means for two to eight profiles (weekday–weekend covariance model)

| **Profile** | **n (assigned)** | **%** | **AvePP** | **ICTSUBJ** | **ICTENQ** | **ICTFEED** | **ICTOUT** | **ICTWKDY** | **ICTWKEND** |
| --- | --- | --- | --- | --- | --- | --- | --- | --- | --- |
| *K = 2* | | | | | | | | | |
| Profile 1 | 44,399 | 53.1 | .923 | 0.45 | 0.47 | 0.56 | 0.45 | 0.26 | 0.23 |
| Profile 2 | 39,264 | 46.9 | .922 | −0.52 | −0.53 | −0.64 | −0.50 | −0.29 | −0.26 |
| *K = 3* | | | | | | | | | |
| Profile 1 | 21,654 | 25.9 | .900 | −0.69 | −0.78 | −0.92 | −0.85 | −0.39 | −0.36 |
| Profile 2 | 16,045 | 19.2 | .884 | 0.84 | 0.89 | 1.10 | 0.82 | 0.51 | 0.45 |
| Profile 3 | 45,964 | 54.9 | .892 | 0.03 | 0.06 | 0.04 | 0.11 | 0.01 | 0.01 |
| *K = 4 (selected solution)* | | | | | | | | | |
| **Profile 1** | **28,056** | **33.5** | **.830** | **−0.41** | **−0.41** | **−0.54** | **−0.26** | **−0.24** | **−0.21** |
| **Profile 2** | **8,463** | **10.1** | **.881** | **−0.85** | **−1.00** | **−1.14** | **−1.38** | **−0.49** | **−0.45** |
| **Profile 3** | **36,571** | **43.7** | **.857** | **0.24** | **0.26** | **0.32** | **0.26** | **0.12** | **0.11** |
| **Profile 4** | **10,573** | **12.6** | **.868** | **0.94** | **1.03** | **1.29** | **0.96** | **0.62** | **0.55** |
| *K = 5* | | | | | | | | | |
| Profile 1 | 960 | 1.1 | .866 | −0.14 | −0.04 | −0.09 | −0.19 | −0.04 | 1.19 |
| Profile 2 | 8,392 | 10.0 | .878 | −0.85 | −1.00 | −1.14 | −1.38 | −0.49 | −0.47 |
| Profile 3 | 36,134 | 43.2 | .854 | 0.25 | 0.27 | 0.33 | 0.27 | 0.12 | 0.09 |
| Profile 4 | 27,775 | 33.2 | .827 | −0.41 | −0.41 | −0.54 | −0.26 | −0.24 | −0.23 |
| Profile 5 | 10,402 | 12.4 | .864 | 0.95 | 1.04 | 1.29 | 0.96 | 0.63 | 0.56 |
| *K = 6* | | | | | | | | | |
| Profile 1 | 8,321 | 9.9 | .877 | −0.85 | −1.00 | −1.14 | −1.38 | −0.49 | −0.47 |
| Profile 2 | 1,268 | 1.5 | .886 | −0.10 | −0.03 | −0.07 | −0.15 | −0.05 | 1.10 |
| Profile 3 | 1,081 | 1.3 | .884 | 0.30 | 0.18 | 0.30 | −0.07 | 1.09 | −0.09 |
| Profile 4 | 27,660 | 33.1 | .825 | −0.41 | −0.41 | −0.54 | −0.25 | −0.25 | −0.23 |
| Profile 5 | 35,296 | 42.2 | .846 | 0.25 | 0.27 | 0.33 | 0.28 | 0.10 | 0.09 |
| Profile 6 | 10,037 | 12.0 | .857 | 0.97 | 1.04 | 1.30 | 0.97 | 0.64 | 0.58 |
| *K = 7* | | | | | | | | | |
| Profile 1 | 7,861 | 9.4 | .876 | −0.86 | −1.01 | −1.15 | −1.41 | −0.51 | −0.48 |
| Profile 2 | 25,672 | 30.7 | .815 | −0.44 | −0.45 | −0.58 | −0.28 | −0.28 | −0.26 |
| Profile 3 | 1,293 | 1.5 | .886 | −0.10 | −0.03 | −0.07 | −0.15 | −0.03 | 1.11 |
| Profile 4 | 1,151 | 1.4 | .874 | 0.30 | 0.19 | 0.30 | −0.07 | 1.10 | −0.05 |
| Profile 5 | 34,875 | 41.7 | .824 | 0.20 | 0.21 | 0.24 | 0.21 | 0.10 | 0.10 |
| Profile 6 | 3,988 | 4.8 | .833 | 1.17 | 1.10 | 1.33 | 0.90 | 1.46 | 1.35 |
| Profile 7 | 8,823 | 10.5 | .777 | 0.69 | 0.86 | 1.13 | 0.92 | 0.02 | 0.01 |
| *K = 8* | | | | | | | | | |
| Profile 1 | 1,301 | 1.6 | .879 | −0.12 | −0.05 | −0.09 | −0.16 | −0.04 | 1.10 |
| Profile 2 | 10,103 | 12.1 | .789 | 0.70 | 0.84 | 1.10 | 0.90 | 0.01 | 0.00 |
| Profile 3 | 1,085 | 1.3 | .870 | 0.26 | 0.16 | 0.28 | −0.07 | 1.07 | −0.11 |
| Profile 4 | 7,148 | 8.5 | .877 | −0.87 | −1.03 | −1.17 | −1.46 | −0.55 | −0.52 |
| Profile 5 | 26,699 | 31.9 | .756 | 0.08 | 0.15 | 0.17 | 0.28 | −0.17 | −0.15 |
| Profile 6 | 9,529 | 11.4 | .743 | 0.37 | 0.23 | 0.27 | −0.05 | 0.87 | 0.79 |
| Profile 7 | 24,280 | 29.0 | .806 | −0.46 | −0.48 | −0.62 | −0.34 | −0.32 | −0.29 |
| Profile 8 | 3,518 | 4.2 | .873 | 1.16 | 1.13 | 1.38 | 0.96 | 1.51 | 1.39 |

*Assignment of the students in the four-profile solution to the five-profile solution (row %)*

| **Four-profile solution** | **K5 Profile 1** | **K5 Profile 2** | **K5 Profile 3** | **K5 Profile 4** | **K5 Profile 5** |
| --- | --- | --- | --- | --- | --- |
| Profile 1 (below-average use) | 1.4 | 0.0 | 0.0 | 98.5 | 0.0 |
| Profile 2 (very low use) | 0.6 | 99.1 | 0.0 | 0.3 | 0.0 |
| Profile 3 (above-average use) | 1.3 | 0.0 | 98.3 | 0.3 | 0.1 |
| Profile 4 (very high use) | 0.4 | 0.0 | 1.6 | 0.0 | 98.0 |

*Note.* Profile means are model-estimated means of the factor scores (factor-score metric; sample SDs: ICTSUBJ 1.03, ICTENQ 0.72, ICTFEED 0.86, ICTOUT 0.80, ICTWKDY 0.70, ICTWKEND 0.66). Profile numbers follow Mplus and differ across solutions. n and % refer to modal assignment (unweighted). AvePP = average posterior probability of the assigned profile.

**Supplementary Table S6.** Three-profile solution: profile characteristics and creative thinking

|  | **Profile 1 (low use)** | **Profile 2 (high use)** | **Profile 3 (average use)** |  |  |
| --- | --- | --- | --- | --- | --- |
| *A. Classification and profile characteristics* | | | | | |
| Weighted share, % (SE) | 31.3 (0.3) | 19.0 (0.3) | 49.7 (0.3) |  |  |
| Students assigned (n) | 21,654 | 16,045 | 45,964 |  |  |
| Average posterior probability | .900 | .884 | .892 |  |  |
| ICTSUBJ (z) | −0.65 | 0.83 | 0.04 |  |  |
| ICTENQ (z) | −1.09 | 1.24 | 0.08 |  |  |
| ICTFEED (z) | −1.05 | 1.29 | 0.07 |  |  |
| ICTOUT (z) | −1.05 | 1.03 | 0.15 |  |  |
| ICTWKDY (z) | −0.55 | 0.73 | 0.02 |  |  |
| ICTWKEND (z) | −0.52 | 0.70 | 0.03 |  |  |
| Girls, % (SE) | 49.7 (0.8) | 49.6 (1.0) | 51.8 (0.6) |  |  |
| Immigrant background, % (SE) | 6.0 (0.3) | 7.1 (0.3) | 7.6 (0.2) |  |  |
| ESCS, mean (SE) | −0.79 (0.02) | −0.43 (0.02) | −0.35 (0.01) |  |  |
| ICTHOME, mean (SE) | −0.09 (0.02) | −0.34 (0.03) | −0.03 (0.01) |  |  |
| ICTSCH, mean (SE) | −0.10 (0.02) | −0.25 (0.03) | −0.04 (0.01) |  |  |
| *B. Mean creative thinking score* | | | | | |
| Model | Profile 1 | Profile 2 | Profile 3 | Overall test D1 (df) |  |
| Pooled (SE) | 26.44 (0.23) | 25.23 (0.27) | 29.16 (0.18) | 91.13 (2, 207.5) |  |
| Country fixed effects (SE) | 26.55 (0.20) | 26.61 (0.29) | 28.56 (0.19) | 36.14 (2, 183.8) |  |
| Pooled + ESCS + gender | 28.06 | 24.98 | 28.34 | — |  |
| Country fixed effects + ESCS + gender | 27.60 | 26.16 | 28.18 | — |  |
| *C. Pairwise differences in score points (d)* | | | | | |
| Model | Profile 1 − Profile 2 | Profile 1 − Profile 3 | Profile 2 − Profile 3 |  |  |
| Pooled | 1.21 (0.10) | −2.72 (−0.22) | −3.93 (−0.31) |  |  |
| Country fixed effects | −0.06 (−0.00)* | −2.02 (−0.16) | −1.96 (−0.16) |  |  |
| Pooled + ESCS + gender | 3.08 (0.25) | −0.28 (−0.02)* | −3.36 (−0.27) |  |  |
| Country fixed effects + ESCS + gender | 1.44 (0.11) | −0.58 (−0.05)* | −2.02 (−0.16) |  |  |
| *D. Assignment of the students in the three-profile solution to the four-profile solution (row %)* | | | | | |
| Three-profile solution | K4 Profile 1 (below-average) | K4 Profile 2 (very low) | K4 Profile 3 (above-average) | K4 Profile 4 (very high) |  |
| Profile 1 (low use) | 60.9 | 39.1 | 0.0 | 0.0 |  |
| Profile 2 (high use) | 0.0 | 0.0 | 34.1 | 65.9 |  |
| Profile 3 (average use) | 32.3 | 0.0 | 67.7 | 0.0 |  |

*Note.* Same model and estimation as the four-profile solution (Table 3; entropy = .770). Estimates in panels A–C are BCH-corrected and weighted by the final student weight; ten plausible values combined with Rubin’s rules; standard errors from the 80 replicate weights. Adjusted models use students with complete ESCS and gender data (N = 81,426; 41 countries). d = difference divided by the pooled within-profile standard deviation (12.54). All differences are significant at p < .05 after Bonferroni correction except those marked *. z = (profile mean − sample mean) / sample SD.

**Supplementary Table S7.** Profile shares by country

| **Country** | **n** | **Profile 1 (below-average use)** | **Profile 2 (very low use)** | **Profile 3 (above-average use)** | **Profile 4 (very high use)** |
| --- | --- | --- | --- | --- | --- |
| ALB | 1,258 | 16.7 | 7.4 | 42.8 | 33.2 |
| AUS | 3,418 | 18.1 | 4.8 | 57.8 | 19.3 |
| BEL | 2,175 | 45.0 | 11.2 | 37.5 | 6.4 |
| BGR | 1,582 | 19.6 | 5.5 | 56.0 | 18.9 |
| BRA | 2,654 | 34.0 | 19.2 | 32.1 | 14.7 |
| BRN | 1,524 | 46.4 | 14.1 | 33.0 | 6.5 |
| CHL | 1,392 | 37.0 | 9.2 | 43.2 | 10.6 |
| CRI | 1,647 | 39.2 | 15.2 | 34.8 | 10.8 |
| CZE | 2,475 | 44.0 | 9.3 | 42.3 | 4.4 |
| DEU | 1,453 | 53.3 | 20.8 | 23.6 | 2.2 |
| DNK | 1,679 | 6.7 | 1.6 | 72.0 | 19.8 |
| DOM | 1,703 | 24.1 | 17.0 | 36.0 | 22.9 |
| ESP | 8,256 | 39.4 | 7.6 | 46.0 | 7.0 |
| EST | 1,641 | 48.1 | 6.7 | 39.8 | 5.3 |
| FIN | 2,533 | 40.9 | 6.8 | 45.0 | 7.3 |
| GRC | 1,680 | 35.5 | 30.0 | 28.9 | 5.6 |
| HKG | 1,542 | 35.4 | 9.5 | 43.3 | 11.8 |
| HRV | 1,618 | 38.9 | 7.3 | 45.7 | 8.1 |
| HUN | 1,847 | 28.9 | 7.0 | 54.0 | 10.1 |
| ISL | 806 | 22.8 | 3.3 | 56.1 | 17.7 |
| ISR | 1,302 | 45.0 | 11.7 | 32.5 | 10.8 |
| ITA | 3,158 | 34.8 | 3.8 | 51.8 | 9.6 |
| JOR | 1,932 | 20.9 | 16.7 | 42.0 | 20.4 |
| KAZ | 5,373 | 17.6 | 7.2 | 47.6 | 27.6 |
| KOR | 1,710 | 35.0 | 30.4 | 28.3 | 6.4 |
| LTU | 1,875 | 30.5 | 4.6 | 50.4 | 14.4 |
| LVA | 1,430 | 39.3 | 4.4 | 49.9 | 6.4 |
| MAC | 1,230 | 38.3 | 9.2 | 45.5 | 7.0 |
| MAR | 1,797 | 28.8 | 28.6 | 33.3 | 9.3 |
| MLT | 785 | 34.4 | 7.0 | 41.6 | 17.0 |
| MYS | 2,141 | 30.3 | 17.2 | 40.1 | 12.4 |
| PAN | 1,097 | 32.7 | 12.8 | 38.9 | 15.6 |
| POL | 1,773 | 44.7 | 7.0 | 41.6 | 6.7 |
| QUR | 932 | 22.5 | 6.3 | 46.1 | 25.1 |
| ROU | 1,976 | 35.2 | 10.3 | 41.2 | 13.3 |
| SAU | 1,898 | 24.0 | 14.3 | 41.0 | 20.7 |
| SGP | 1,807 | 29.7 | 5.3 | 52.2 | 12.8 |
| SVK | 1,502 | 33.9 | 8.8 | 47.0 | 10.3 |
| SVN | 1,748 | 47.4 | 11.5 | 36.7 | 4.5 |
| TAP | 1,563 | 43.4 | 19.6 | 31.8 | 5.1 |
| THA | 2,301 | 17.3 | 12.8 | 47.4 | 22.6 |
| URY | 1,450 | 35.5 | 11.5 | 39.2 | 13.8 |

*Note.* Weighted (final student weight), BCH-corrected percentages of students in each profile; rows sum to 100. n = students in the analysis sample. Because measurement invariance of the ICT-use factors across countries was not tested, differences between countries are descriptive and should not be interpreted as differences in the same construct.

**Supplementary Table S8.** Creative thinking by ICT use profile within ESCS quartiles and gender

| **Subgroup** | **Model** | **Profile 1** | **Profile 2** | **Profile 3** | **Profile 4** |  |
| --- | --- | --- | --- | --- | --- | --- |
| *A. Mean creative thinking score* | | | | | | |
| All students (n = 83,663) | Pooled | 30.0 | 23.5 | 28.1 | 24.4 |  |
|  | Country fixed effects | 29.2 | 24.4 | 27.9 | 26.1 |  |
| ESCS Q1 (lowest) (n = 12,898) | Pooled | 22.0 | 18.2 | 19.3 | 17.2 |  |
|  | Country fixed effects | 21.6 | 18.6 | 19.1 | 17.8 |  |
| ESCS Q2 (n = 19,301) | Pooled | 27.6 | 23.8 | 25.6 | 21.9 |  |
|  | Country fixed effects | 26.9 | 23.6 | 25.8 | 23.5 |  |
| ESCS Q3 (n = 22,320) | Pooled | 31.8 | 27.9 | 29.3 | 25.5 |  |
|  | Country fixed effects | 31.1 | 27.2 | 29.5 | 27.2 |  |
| ESCS Q4 (highest) (n = 26,910) | Pooled | 37.9 | 33.8 | 35.6 | 32.6 |  |
|  | Country fixed effects | 37.4 | 33.1 | 35.7 | 34.2 |  |
| Boys (n = 41,726) | Pooled | 29.1 | 23.4 | 26.5 | 22.7 |  |
|  | Country fixed effects | 28.3 | 23.9 | 26.4 | 24.3 |  |
| Girls (n = 41,934) | Pooled | 30.8 | 23.7 | 29.6 | 26.2 |  |
|  | Country fixed effects | 29.8 | 25.0 | 29.4 | 28.1 |  |
| All students, senate weights | Pooled | 31.65 | 24.55 | 29.83 | 25.39 |  |
| *B. Pairwise differences: Cohen's d; ns = not significant after Bonferroni adjustment (p ≥ .05)* | | | | | | |
| **Subgroup** | **Model** | **1 − 2** | **1 − 3** | **1 − 4** | **2 − 3** | **2 − 4 / 3 − 4** |
| All students | Pooled | 0.52 | 0.16 | 0.45 | −0.36 | −0.07 *^ns^* / 0.29 |
|  | Country fixed effects | 0.39 | 0.10 | 0.25 | −0.28 | −0.14 / 0.14 |
| ESCS Q1 (lowest) | Pooled | 0.30 | 0.22 | 0.39 | −0.08 *^ns^* | 0.08 *^ns^* / 0.17 |
|  | Country fixed effects | 0.24 | 0.20 | 0.31 | −0.04 *^ns^* | 0.07 *^ns^* / 0.11 *^ns^* |
| ESCS Q2 | Pooled | 0.31 | 0.15 | 0.46 | −0.15 | 0.15 *^ns^* / 0.30 |
|  | Country fixed effects | 0.26 | 0.08 *^ns^* | 0.27 | −0.18 | 0.01 *^ns^* / 0.19 |
| ESCS Q3 | Pooled | 0.32 | 0.20 | 0.51 | −0.12 *^ns^* | 0.19 / 0.31 |
|  | Country fixed effects | 0.32 | 0.13 | 0.32 | −0.19 | 0.00 *^ns^* / 0.19 |
| ESCS Q4 (highest) | Pooled | 0.33 | 0.19 | 0.43 | −0.14 *^ns^* | 0.10 *^ns^* / 0.24 |
|  | Country fixed effects | 0.34 | 0.14 | 0.25 | −0.20 | −0.09 *^ns^* / 0.11 *^ns^* |
| Boys | Pooled | 0.45 | 0.21 | 0.51 | −0.25 | 0.06 *^ns^* / 0.30 |
|  | Country fixed effects | 0.35 | 0.15 | 0.32 | −0.20 | −0.03 *^ns^* / 0.16 |
| Girls | Pooled | 0.57 | 0.10 | 0.37 | −0.48 | −0.20 / 0.27 |
|  | Country fixed effects | 0.39 | 0.03 *^ns^* | 0.14 | −0.36 | −0.25 / 0.10 |

*Note.* Profile 1 = below-average use; Profile 2 = very low use; Profile 3 = above-average use; Profile 4 = very high use. BCH-corrected means weighted by the final student weight (one decimal); ten plausible values; standard errors from the 80 replicate weights. ESCS quartiles are weighted quartiles of the pooled sample. d = difference divided by the pooled within-profile standard deviation of the full sample (12.41). Senate weights: rescaled to 5,000 per country.

**Supplementary Table S9.** Random forest models: additional results

|  |  |  |  |  |
| --- | --- | --- | --- | --- |
| *A. Cross-validated R² by sample and predictor set (mean over ten plausible values)* | | | | |
| **Sample** | **Predictor set** | **n** | **Weighted R²** | **Unweighted R²** |
| Main (imputed) | Profile | 82,016 | .022 | −.007 |
| Main (imputed) | Six factor scores | 82,016 | .165 | .174 |
| Main (imputed) | Background + availability | 82,016 | .261 | .228 |
| Main (imputed) | Background + availability + profile | 82,016 | .272 | .241 |
| Main (imputed) | Background + availability + six factor scores | 82,016 | .332 | .314 |
| Main (imputed) | Background + availability + profile + six factor scores | 82,016 | .332 | .315 |
| Main (imputed) | Background + availability + posterior probabilities | 82,016 | .262 | .234 |
| Main (imputed) | Background + availability + profile (random draw) | 82,016 | .270 | .240 |
| Main (imputed) | Background + availability + country | 82,016 | .360 | .363 |
| Main (imputed) | Background + availability + profile + country | 82,016 | .369 | .370 |
| Main (imputed) | Background + availability + six factor scores + country | 82,016 | .412 | .414 |
| Complete cases | Profile | 68,576 | .024 | .000 |
| Complete cases | Six factor scores | 68,576 | .187 | .180 |
| Complete cases | Background + availability | 68,576 | .251 | .215 |
| Complete cases | Background + availability + profile | 68,576 | .261 | .227 |
| Complete cases | Background + availability + six factor scores | 68,576 | .334 | .308 |
| Complete cases | Background + availability + profile + six factor scores | 68,576 | .335 | .309 |
| Complete cases | Background + availability + country | 68,576 | .350 | .341 |
| Complete cases | Background + availability + profile + country | 68,576 | .360 | .348 |
| Complete cases | Background + availability + six factor scores + country | 68,576 | .412 | .401 |
| Item-level subsample | Profile | 47,059 | .022 | .005 |
| Item-level subsample | Six factor scores | 47,059 | .168 | .155 |
| Item-level subsample | 40 items | 47,059 | .273 | .265 |
| Item-level subsample | Background + availability | 47,059 | .223 | .184 |
| Item-level subsample | Background + availability + profile | 47,059 | .230 | .195 |
| Item-level subsample | Background + availability + six factor scores | 47,059 | .308 | .275 |
| Item-level subsample | Background + availability + 40 items | 47,059 | .360 | .336 |
| Item-level subsample | Background + availability + profile + 40 items | 47,059 | .360 | .336 |
| *B. Change in weighted R² in the item-level subsample (students who answered all 40 items; n = 47,059)* | | | | |
|  | Profile added to background + availability |  | .0072 [.0056, .0093] | 10/10 PVs with a gain |
|  | Six factor scores added to background + availability |  | .0851 [.0765, .0903] | 10/10 PVs with a gain |
|  | 40 items added to background + availability |  | .1372 [.1294, .1430] | 10/10 PVs with a gain |
|  | 40 items instead of profile (with background + availability) |  | .1300 [.1226, .1359] | 10/10 PVs with a gain |
|  | 40 items instead of six factor scores (with background + availability) |  | .0521 [.0466, .0550] | 10/10 PVs with a gain |
|  | Profile added to background + availability + 40 items |  | .0001 [−.0010, .0016] | 5/10 PVs with a gain |
|  | 40 items instead of profile (ICT use only) |  | .2505 [.2439, .2551] | 10/10 PVs with a gain |
|  | Six factor scores instead of profile (ICT use only) |  | .1460 [.1370, .1510] | 10/10 PVs with a gain |
| *C. Three-profile solution: change in weighted R², main analysis* | | | | |
|  | Profile added to background + availability |  | .0066 [.0047, .0092] | 10/10 PVs with a gain |
|  | Six factor scores added to background + availability |  | .0760 [.0685, .0821] | 10/10 PVs with a gain |
|  | Six factor scores instead of profile (with background + availability) |  | .0693 [.0634, .0729] | 10/10 PVs with a gain |
|  | Profile added to background + availability + six factor scores |  | .0006 [−.0010, .0019] | 8/10 PVs with a gain |
|  | Six factor scores added to background + availability + profile |  | .0700 [.0653, .0740] | 10/10 PVs with a gain |
|  | Six factor scores instead of profile (ICT use only) |  | .1638 [.1573, .1699] | 10/10 PVs with a gain |
|  | Posterior probabilities instead of modal profile |  | .0078 [.0032, .0098] | 10/10 PVs with a gain |
|  | Random profile draw instead of modal profile |  | −.0003 [−.0010, .0008] | 2/10 PVs with a gain |
|  | Country added to background + availability |  | .0985 [.0937, .1014] | 10/10 PVs with a gain |
|  | Profile added to background + availability + country |  | .0073 [.0045, .0106] | 10/10 PVs with a gain |
|  | Six factor scores added to background + availability + country |  | .0561 [.0511, .0615] | 10/10 PVs with a gain |
|  | Country added to background + availability + six factor scores |  | .0787 [.0739, .0807] | 10/10 PVs with a gain |
| *D. Permutation importance in held-out schools, main analysis (% increase in weighted MSE; mean [min, max] over PVs)* | | | | |
| Background + availability + profile | ESCS |  | 55.3 [53.0, 57.2] | positive in all PVs |
|  | Profile (modal) |  | 3.4 [2.8, 3.8] | positive in all PVs |
|  | Gender (girl) |  | 3.0 [2.6, 3.5] | positive in all PVs |
|  | ICT availability outside school (ICTHOME) |  | 2.3 [2.1, 2.4] | positive in all PVs |
|  | ICT availability at school (ICTSCH) |  | 1.8 [1.6, 2.0] | positive in all PVs |
|  | Immigrant background |  | 0.7 [0.4, 0.9] | positive in all PVs |
|  | Background (gender, ESCS, immigrant background) [group] |  | 57.7 [55.6, 60.2] | positive in all PVs |
|  | Availability (ICTHOME, ICTSCH) [group] |  | 4.2 [3.7, 4.5] | positive in all PVs |
| Background + availability + six factor scores | ESCS |  | 47.5 [45.9, 49.4] | positive in all PVs |
|  | ICTOUT (factor score) |  | 7.4 [6.4, 8.0] | positive in all PVs |
|  | ICTWKDY (factor score) |  | 7.0 [6.4, 7.7] | positive in all PVs |
|  | ICTFEED (factor score) |  | 4.7 [4.1, 5.3] | positive in all PVs |
|  | ICTWKEND (factor score) |  | 3.4 [2.7, 3.9] | positive in all PVs |
|  | ICTSUBJ (factor score) |  | 2.1 [1.7, 2.6] | positive in all PVs |
|  | ICTENQ (factor score) |  | 1.6 [1.3, 1.8] | positive in all PVs |
|  | Gender (girl) |  | 1.4 [1.1, 1.6] | positive in all PVs |
|  | ICT availability outside school (ICTHOME) |  | 0.6 [0.4, 0.7] | positive in all PVs |
|  | ICT availability at school (ICTSCH) |  | 0.4 [0.2, 0.6] | positive in all PVs |
|  | Immigrant background |  | 0.3 [0.2, 0.4] | positive in all PVs |
|  | Background (gender, ESCS, immigrant background) [group] |  | 48.7 [47.5, 50.0] | positive in all PVs |
|  | Six factor scores [group] |  | 28.5 [26.7, 30.5] | positive in all PVs |
|  | Availability (ICTHOME, ICTSCH) [group] |  | 1.2 [1.1, 1.3] | positive in all PVs |
| Background + availability + profile + six factor scores | ESCS |  | 46.9 [45.3, 48.8] | positive in all PVs |
|  | ICTOUT (factor score) |  | 7.6 [6.9, 8.6] | positive in all PVs |
|  | ICTWKDY (factor score) |  | 6.8 [6.0, 7.4] | positive in all PVs |
|  | ICTFEED (factor score) |  | 4.4 [3.9, 4.9] | positive in all PVs |
|  | ICTWKEND (factor score) |  | 3.2 [2.6, 3.8] | positive in all PVs |
|  | ICTSUBJ (factor score) |  | 2.0 [1.2, 2.5] | positive in all PVs |
|  | ICTENQ (factor score) |  | 1.5 [1.3, 1.7] | positive in all PVs |
|  | Gender (girl) |  | 1.4 [1.2, 1.7] | positive in all PVs |
|  | ICT availability outside school (ICTHOME) |  | 0.6 [0.4, 0.7] | positive in all PVs |
|  | ICT availability at school (ICTSCH) |  | 0.4 [0.2, 0.5] | positive in all PVs |
|  | Immigrant background |  | 0.3 [0.2, 0.4] | positive in all PVs |
|  | Profile (modal) |  | 0.1 [−0.1, 0.3] | not positive in all PVs |
|  | Background (gender, ESCS, immigrant background) [group] |  | 48.0 [46.1, 49.7] | positive in all PVs |
|  | Six factor scores [group] |  | 26.3 [25.2, 28.3] | positive in all PVs |
|  | Availability (ICTHOME, ICTSCH) [group] |  | 1.1 [0.9, 1.3] | positive in all PVs |

*Note.* R² in held-out schools from five-fold cross-validation (schools assigned to folds within countries); weighted R² uses the final student weight. Unweighted R² below zero means that the model predicted held-out students less accurately than their unweighted mean. Background = gender, ESCS and immigrant background; availability = ICTHOME and ICTSCH. Permutation importance: increase in the weighted mean squared prediction error when a predictor (or a group of predictors) is permuted within the held-out fold, as a percentage of that error; importance is shared among correlated predictors and does not indicate direction. In the item-level subsample, the most important single items were IC173Q04 (use in computer science lessons; 5.1%) and IC174Q02 (writing or editing text for a school assignment; 3.5%); students without computer science lessons are not part of this subsample and country was not a predictor, so the importance of IC173Q04 may partly reflect curricular differences between countries.
